# Sexual dimorphism in bidirectional SR-mitochondria crosstalk in ventricular cardiomyocytes

**DOI:** 10.1007/s00395-023-00988-1

**Published:** 2023-05-03

**Authors:** Richard T. Clements, Radmila Terentyeva, Shanna Hamilton, Paul M. L. Janssen, Karim Roder, Benjamin Y. Martin, Fruzsina Perger, Timothy Schneider, Zuzana Nichtova, Anindhya S. Das, Roland Veress, Beth S. Lee, Do-Gyoon Kim, Gideon Koren, Matthew S. Stratton, Gyorgy Csordas, Federica Accornero, Andriy E. Belevych, Sandor Gyorke, Dmitry Terentyev

**Affiliations:** 1 Department of Biomedical and Pharmaceutical Sciences, University of Rhode Island College of Pharmacy, Kingston, RI USA; 2 Department of Medicine, Providence VAMC and Brown University, Providence, RI USA; 3 Department of Physiology and Cell Biology, The Ohio State University, 460 Medical Center Dr, Columbus, OH 43210 USA; 4 Dorothy M. Davis Heart and Lung Research Institute, College of Medicine, The Ohio State University Wexner Medical Center, Columbus, OH USA; 5 Department of Medicine, Cardiovascular Research Center, Rhode Island Hospital, The Warren Alpert Medical School of Brown University, Providence, RI USA; 6 Department of Pathology, Anatomy and Cell Biology, MitoCare Center, Thomas Jefferson University, Philadelphia, PA USA; 7 Division of Orthodontics, College of Dentistry, The Ohio State University, Columbus, OH USA

**Keywords:** Cardiovascular diseases, Mitochondria, Sarcoplasmic reticulum Ca^2+^ release, Sexual dimorphism, Oxidative stress, COX7RP

## Abstract

Calcium transfer into the mitochondrial matrix during sarcoplasmic reticulum (SR) Ca^2+^ release is essential to boost energy production in ventricular cardiomyocytes (VCMs) and match increased metabolic demand. Mitochondria from female hearts exhibit lower mito-[Ca^2+^] and produce less reactive oxygen species (ROS) compared to males, without change in respiration capacity. We hypothesized that in female VCMs, more efficient electron transport chain (ETC) organization into supercomplexes offsets the deficit in mito-Ca^2+^ accumulation, thereby reducing ROS production and stress-induced intracellular Ca^2+^ mishandling. Experiments using mitochondria-targeted biosensors confirmed lower mito-ROS and mito-[Ca^2+^] in female rat VCMs challenged with β-adrenergic agonist isoproterenol compared to males. Biochemical studies revealed decreased mitochondria Ca^2+^ uniporter expression and increased supercomplex assembly in rat and human female ventricular tissues vs male. Importantly, western blot analysis showed higher expression levels of COX7RP, an estrogen-dependent supercomplex assembly factor in female heart tissues vs males. Furthermore, COX7RP was decreased in hearts from aged and ovariectomized female rats. COX7RP overexpression in male VCMs increased mitochondrial supercomplexes, reduced mito-ROS and spontaneous SR Ca^2+^ release in response to ISO. Conversely, shRNA-mediated knockdown of COX7RP in female VCMs reduced supercomplexes and increased mito-ROS, promoting intracellular Ca^2+^ mishandling. Compared to males, mitochondria in female VCMs exhibit higher ETC subunit incorporation into supercomplexes, supporting more efficient electron transport. Such organization coupled to lower levels of mito-[Ca^2+^] limits mito-ROS under stress conditions and lowers propensity to pro-arrhythmic spontaneous SR Ca^2+^ release. We conclude that sexual dimorphism in mito-Ca^2+^ handling and ETC organization may contribute to cardioprotection in healthy premenopausal females.

## Introduction

The incidence of all causes of cardiovascular disease (CVD) is much lower in premenopausal females in comparison to males of the same age [52]. However, later in life CVD becomes as prevalent in females as males, often resulting in sudden cardiac death due to ventricular arrhythmias [40]. The major driver of cardiac dysfunction in old age has been ascribed to defective mitochondrial function, leading to impaired metabolic flexibility and subsequent aberrant stress responses [35]. Defective electron transport results in increased production of reactive oxygen species (ROS) by mitochondria, which can impair function of numerous enzymes and ion channels in cardiomyocytes [15, 19, 21]. Interestingly, in younger males, mitochondria are known to produce more ROS than mitochondria of younger females [14, 51], without differences in respiration capacity [29, 30]. However, the exact mechanisms underlying this fundamental difference between males and females, which may explain sexual dimorphism in rates of CVD, are yet to be defined.

Cardiac contractility relies on tightly controlled release of Ca^2+^ from the sarcoplasmic reticulum (SR) mediated by cardiac ryanodine receptors type 2 (RyR2) [6]. The amplitude of SR Ca^2+^ release in ventricular cardiomyocytes (VCMs) during systole determines the strength of contractile apparatus activation, while timely cessation of RyR2 activity and effective clearance of Ca^2+^ from the cytosol by SR Ca^2+^ ATPase (SERCA2a) in diastole promotes relaxation after each beat. Oxidative modifications of the SR Ca^2+^ handling machinery profoundly impair intracellular Ca^2+^ cycling by impeding SERCA2a-mediated Ca^2+^ uptake and facilitating untimely RyR2 channel opening [19]. This leads not only to the loss of SR Ca^2+^ content, but also to generation of spontaneous diastolic Ca^2+^ waves (SCWs) that promote disturbances in sarcolemma membrane potential in the form of delayed- and early-afterdepolarizations. These disturbances underlie pro-arrhythmic triggered activity at the whole heart level. The risk of pro-arrhythmic behavior profoundly escalates under stress conditions typically accompanied by β-adrenergic stimulation. Beta-adrenergic stimulation increases spontaneous SR Ca^2+^ release and coincidentally increases mito-ROS production [9]. Recently we have shown that RyR2-mediated SR Ca^2+^ release increases transfer of Ca^2+^ to the mitochondrial matrix via mitochondrial Ca^2+^ uniporter (MCU). This increase contributes to mito-ROS production in VCMs challenged with isoproterenol (ISO), a selective β-adrenergic agonist [23]. Furthermore, ROS produced by mitochondria lead to an increase in RyR2 oxidation and thereby channel activity, promoting pro-arrhythmic spontaneous SCWs [15, 22, 23].

Cardiac contraction is a high-energy demanding process. Mitochondria are the major source of energy in VCMs and their ability to generate ATP must be tightly matched to rapidly changing metabolic demands [16, 21]. In addition to sensing ATP/ADP ratio, mitochondrial function can be tuned by changes in [Ca^2+^] entering matrix via MCU. An increase in mitochondrial matrix [Ca^2+^] activates several dehydrogenases stimulating NADH and FAD_2_ production that fuel electron transport chain (ETC). Interestingly, studies using mouse models with loss-of-MCU function demonstrated no change in basal cardiac function but showed significant loss of responsiveness to adrenergic stimulation [38, 45]. This suggests that MCU-mediated mito-Ca^2+^ uptake has a limited yet important function to boost energy production during stress as an integral part of the fight-or-flight response. Notably, it has been demonstrated that mito-[Ca^2+^] is significantly lower in female VCMs than males [2]. However, cardiac mitochondrial respiration capacity [29, 30] and cardiac output [25, 27] are comparable between males and females, suggesting that SR Ca^2+^ release-bioenergetics coupling is fundamentally different between sexes, and that these differences may play a key role in lowering cardiovascular risk in premenopausal females.

In order to provide similar metabolic output to males, it is reasonable to hypothesize that mitochondria from female VCMs likely have more efficient electron transport to compensate for the reduction of matrix [Ca^2+^] [2]. An additional benefit of a more efficient ETC organization would include reduced levels of ROS [14]. It was recently established that elements of the ETC residing in mitochondrial cristae can form large multimolecular supercomplexes, which substantially increase electron transport efficiency [12]. A group of proteins involved in facilitation of supercomplex assembly has recently been identified [3]. Of particular interest is the supercomplex assembly factor, COX7RP (also known as Cox7a2l and SCAFI), given its expression is dependent on the sex hormone estrogen [33, 53]. However, whether potential differences in mitochondrial supercomplexes and COX7RP expression levels contribute to reduced mito-ROS production in female VCMs, thus exerting a stabilizing influence on the SR Ca^2+^ release, remains unknown.

In the present study we investigated the role of sex and COX7RP-dependent changes in mitochondrial supercomplex assembly, mito-ROS production, and intracellular Ca^2+^ homeostasis in VCMs under conditions mimicking stress. Our results demonstrate a higher degree of ETC organization into supercomplexes and reduced mito-[Ca^2+^] uptake in female VCMs vs males. This leads to reduced mito-ROS production and less pro-arrhythmic SCWs due to reduced RyR2 oxidation under β-adrenergic stimulation. Our data suggest that sexual dimorphism in mitochondrial Ca^2+^ handling and ETC organization may contribute to cardioprotection in healthy premenopausal females.

## Methods

### Study animals

All procedures involving animals were performed following the National Institutes of Health Guide for the Care and Use of Laboratory Animals published by the US National Institutes of Health (NIH Publication No. 85-23, revised 2011) and were approved by the Institutional Animal Care and Use Committees of The Ohio State University, University of Rhode Island and Rhode Island Hospital. Two-month-old male and female Sprague–Dawley rats (control) were purchased from Charles River Laboratories (Wilmington, MA, United States) and heart tissue samples and isolated ventricular myocytes were studied at 3–4 month of age. In addition seven 6 month old Sprague–Dawley rats were bilaterally ovariectomized (OVX) and additional five rats received a sham surgery (Sham) at Harlan Laboratories Inc. Indianapolis, IN, USA. These rats were used 3 months after surgery to obtain heart tissue samples. To study potential sex differences in intracellular Ca^2+^ homeostasis, mito-ROS and mito-Ca^2+^ 22-month-old F344 rats of both sexes were obtained from NIH aged rat colony.

### Human samples

Human left ventricular tissues were obtained from human organ donors as previously described [20, 39]. These non-failing hearts were from those organ donors who had no history of AF and/or major cardiovascular diseases but not used for heart transplantation. No one involved in the study had any involvement in clinical decision making regarding suitability of donor heart use. The studies were approved by the Human Study Committee of The Ohio State University. The hearts were evaluated by Lifeline of Ohio Organ Procurement Team, an entity not involved in the study, and were initially deemed potentially suitable for transplantation. The donor’s files do not contain specific entry why a specific heart was not used. According to the coordinators, the most common reasons for not using a heart is that it was too small/gender disparity, or the potential presence of a viral infection. Demographic data of donors from whom tissue samples were obtained are described in Table 1.

**Table 1:** De-identified demographic data of human organ donors

Sample	Sex	Age (years)	Race
1	Male	36	Caucasian
2	Male	22	African American
3	Male	44	African American
4	Male	26	Caucasian
5	Male	60	Caucasian
6	Male	51	Unknown
7	Male	62	Unknown
8	Female	43	Caucasian
9	Female	43	African American
10	Female	68	Caucasian
11	Female	38	Caucasian
12	Female	52	Unknown
13	Female	19	Caucasian
14	Female	24	African American
15	Female	26	Caucasian
16	Female	28	African American
17	Female	29	Caucasian
18	Female	30	Caucasian
19	Female	31	Caucasian
20	Female	32	Caucasian
21	Female	34	African American
22	Female	34	Caucasian
23	Female	60	Caucasian
24	Female	61	Caucasian
25	Female	62	Caucasian
26	Female	62	N/A
27	Female	63	Caucasian
28	Female	64	Caucasian
29	Female	65	Caucasian
30	Female	67	Caucasian
31	Female	69	Caucasian
32	Female	72	Caucasian

### Molecular biology

The mitochondrial matrix Ca^2+^ sensor mtRCamp1h was constructed by fusing cytochrome C oxidase subunit IV at the N-terminus of RCamp1h coding region as described previously [1, 23]. Mito-GFP was constructed by fusing a mitochondrial targeting sequence to GFP. The MLS-HyPer-7 sensor was a gift from Vsevolod Belousov [42]. Adenoviruses carrying biosensor constructs were generated using the ViraPower Gateway expression system (Thermo Fisher Scientific, Waltham, MA, USA) [24]. COX7RP-FLAG and shRNA COX7RP adenoviruses were purchased from Vigene Biosciences and Vector Biolabs, respectively.

### Myocyte isolation and cell culture

To isolate ventricular cardiomyocytes (VCMs), rats were injected with pentobarbital (120 mg/kg) as a terminal procedure, and hearts removed via bilateral thoracotomy. Hearts were placed in ice-cold Tyrode’s solution and mounted on a Langendorff apparatus before retrograde perfusion with Tyrode’s solution (in mmol/L: 140 NaCl, 5.4 KCl, 1 MgCl_2_, 10 HEPES, 5.6 glucose, pH 7.3) containing collagenase II (Worthington Biochemical Corp., Lakewood, NJ, USA) at 37 °C for 15–20 min. Atria were removed and ventricles were minced and placed in a 37 °C water bath shaker in collagenase solution. Isolated VCMs were plated in serum onto laminin-coated glass coverslips in 24-well plates (Medium 199 [Thermo Fisher Scientific] supplemented with 25 mmol/L NaHCO_3_, 10 mmol/L HEPES, 5 mmol/L creatine, 5 mmol/L taurine, 10 μg/mL penicillin, 10 μg/mL streptomycin, and 10 μg/mL gentamycin; pH 7.3). Unattached cells were removed after 1 h. Attached VCMs were infected with adenoviruses described below at multiplicity of infection (MOI) of 10. Myocytes were cultured at 37 °C in 95% air, 5% CO_2_ for 36–44 h before analysis.

### Transmission electron microscopy

For perfusion fixation, animals were heparinized, then euthanized by pentobarbital (120 mg/kg) injection. The hearts were cannulated through the aorta for retrograde perfusion on a Langendorff apparatus. First, the hearts were perfused by cold Ca^2+^-free Tyrode solution (in mmol/L: 140 NaCl, 5.4 KCl, 1 MgCl_2_, 10 HEPES, 5.6 glucose (pH 7.3) for ~ 5 min (until the heart stops beating). This is followed by perfusion with 2.5% glutaraldehyde in 0.1 M Na^+^-cacodylate buffer (pH 7.4) for 5 min at room temperature. Then the heart was immersed into the fixative for an additional 40 min before the apex was removed to cut the LV anterior wall open longitudinally along the anterior wall. After additional 30 min at room temperature, the heart in the excessive fixative was put on ice in individually labeled sealed vials, and shipped to the MitoCare Center Electron Microscopy Facility at Thomas Jefferson University, for further processing. There, small pieces from the LV wall were cut (~ 1 mm^3^), overnight postfixed in 4 °C in 2% osmium tetroxide partially reduced by 0.8% K_4_Fe(CN)_6_ in 0.15 mol Na^+^-cacodylate buffer. Samples were further contrasted en bloc with 1% aqueous uranylacetate, dehydrated in graded series of acetone or propyleneoxide, embedded in Spurr’s or Epon (Embed 812) resin. Longitudinal, ultrathin sections (65–80 nm) were cut from the resin-embedded blocks with a diamond knife (Diatome-US, USA) using a Leica UCT ultramicrotome and caught on copper grid covered with formvar film or a Gilder parallel bar grid (100 mesh). Images of longitudinal oriented cardiomyocytes were obtained via an FEI Tecnai 12 TEM fitted with an AMT XR-111 10.5 Mpx or (later) AMT BioSprint 12 12 Mpx CCD camera at 3200–15,000 × magnification (80 kV). Methods for analyzing mitochondria morphology were adapted from Khalifa et al. [30]. TEM images were uniformly adjusted for brightness/contrast and mitochondria were manually selected by the freehand tool using ImageJ software (ImageJ, NIH). Individual mitochondria were analyzed for area, perimeter, aspect ratio (the ratio between major and minor axes), and form factor. Form factor (F), a measure of mitochondrial length was determined for each image using the equation F = (perimeter^2^/(4*π* × area). Mitochondria density was expressed as the percentage of cell area occupied by mitochondrion.

### Measurements of intracellular Ca^2+^ transients

Confocal imaging was performed using a Leica SP8 confocal microscope equipped with a 63 × 1.4 numerical aperture oil objective. Cultured rat VCMs were loaded with Fluo-3 AM (Invitrogen) at room temperature for 10 min in Ca^2+^-free Tyrode’s solution, followed by a 10 min wash in Tyrode’s solution containing 1 mmol/L Ca^2+^. Myocytes were perfused with Tyrode’s solution (1 mmol/L Ca^2+^) during recordings, at room temperature. Myocytes were paced via field stimulation at 1 Hz with platinum electrodes. To test for the propensity of spontaneous Ca^2+^ waves (SCWs), isoproterenol-treated (50 nmol/L) VCMs were paced for 20 s and latency between the last pacing stimulus and the SCW was calculated. Caffeine (10 mmol/L) was applied at the end of recording to assess SR Ca^2+^ content. Fluo-3AM was excited at 488 nm and fluorescence emission was collected at 500–550 nm wavelengths in line scan mode at 200 Hz sampling rate. Cytosolic Ca^2+^ transient amplitude is presented as ΔF/F_0_, where F_0_ is basal fluorescence and ΔF = F–F_0_.

### Measurements of mitochondrial matrix Ca^2+^

Myocytes were infected with mtRCamp1h adenovirus ± COX7RP or COX7RP-shRNA adenovirus and cultured for 36–44 h [20]. mtRCamp1h was excited using 543 nm line of HeNe laser and fluorescence emission collected at 560–660 nm wavelengths, measured in the x–y mode at 400 Hz sampling rate. After pacing, VCMs were washed in Ca^2+^-free Tyrode’s solution before permeabilization with saponin (0.001%). Tyrode’s solution was replaced with internal recording solution. The intracellular solution contained (mmol/L): 120 K^+^ aspartate, 20 KCl, 0.81 MgCl_2_, 1 KH_2_PO_4_, 3 MgATP, 10 phosphocreatine, 20 HEPES (pH 7.2) and 5 U/mL creatine phosphokinase. Internal solution was supplemented with cytochalasin D (10 μmol/L) to inhibit contraction and Ca^2+^ buffer EGTA (2 mmol/L) to obtain minimum mtRCamp1h fluorescence [20]. Maximum fluorescence was achieved by application of Ca^2+^ (20 μmol/L) in EGTA-free solution. Using the equation mito-[Ca^2+^] = Kd × (F − Fmin)/(Fmax − F), where Kd of mtRCamp1h = 1.3 µmol/L for Ca^2+^, fluorescence was converted to mito-[Ca^2+^] for each VCM. Analysis parameters included baseline mtRCamp1h mito-[Ca^2+^] (μmol/L) in resting VCMs; peak mito-[Ca^2+^] and the time to peak amplitude (s) in VCMs undergoing periodic 1 Hz field stimulation [24]. The rate of mito-Ca^2+^ decay (1/τ, s^−1^) was derived from single exponential fit of VCM mito-[Ca^2+^] decrease upon cessation of pacing.

### Measurements of mitochondrial matrix ROS

The ratiometric MLS-HyPer7 probe was excited using 405 nm and 488 nm laser lines. Fluorescence emission was collected at 520–540 nm wavelengths, measured in the x–y mode at 400 Hz sampling rate. Minimum fluorescence was obtained by application of dithiothreitol (DTT, 5 mmol/L), and maximum fluorescence was obtained by application of 2,2′-dithiodipyridine (DTDP, 200 µmol/L). Data are presented as a percentage of ΔF/ΔFmax where ΔF = F − Fmin and ΔFmax = Fmax − Fmin [24].

### Western blot analysis of cardiac mitochondrial proteins

Tissue samples or cultured rat VCMs were lysed in lysis buffer from Cell Signaling (catalogue no. 9803S), supplemented with phosphatase (Calbiochem, San Diego, CA, USA; catalogue no. 524625) and protease inhibitor cocktails (Sigma; catalogue no. P8340) as described previously [31]. Samples (20–30 μg of proteins) were resolved on a 4–20% gel via SDS‐PAGE, transferred onto nitrocellulose membranes, and probed with anti-COX7a2L (COX7RP), anti-MCU, anti-Hsp60, anti-GAPDH antibodies and subsequently probed with a goat anti‐mouse, goat anti‐rabbit secondary (Promega; Madison, WI, USA). Blots were developed with ECL (Bio‐Rad Laboratories, Hercules, CA, USA; catalogue no. 1705061) and quantified and analyzed using ImageJ (NIH, Bethesda, MD, USA;) and Origin, version 8 (OriginLab Corp., Northampton, MA, USA). COX7RP and MCU signals were normalized to Hsp60 loading controls. List of antibodies used is present in Table 2.

**Table 2:** Antibodies used in this study

Antibody/kit	Species of used sample	Source	Identifier
Anti-COX7a2L	H9C2,Rat and Human	MilliporeSigma	Cat# SAB1303595
Anti-RyR2	Rat and Human	Alomone	Cat# ARR-002
Anti-MCU	Rat and Human	MilliporeSigma	Cat# HPA016480
Anti-COX IV	Rat and Human	Abcam	Cat# ab16056
Anti-UQCRFS1	Rat and Human	Abcam	Cat# ab14746
Anti-NDUFA9	Rat and Human	Abcam	Cat# ab14713
Anti-Hsp60	H9C2, Rat and Human	Cell Signaling	Cat# 12165T
Anti-GAPDH	H9C2, Rat and Human	Abcam	Cat# ab8245
Anti-Rabbit IgG(H+L),HRP	H9C2, Rat and Human	Promega	Cat# W4011
Anti-Mouse IgG(H+L),HRP	H9C2, Rat and Human	Promega	Cat# W4021
Oxidized Protein Western blot kit	Rat	Abcam	Cat# ab178020
Anti-MnSOD	Rat	Millipore	Cat # 06-984
Anti-Peroxiredoxin 5	Rat	Abcam	Cat # ab180123

### Western blot analysis of H9c2 cells

H9c2 cells were plated in 6-well plates and infected with COX7RP-FLAG and shRNA COX7RP adeno-viruses. At 48 h after infection, cells were lysed in lysis buffer from Cell Signaling (Cat # 9803S), supplemented with phosphatase (Calbiochem, Cat#524,625) and protease inhibitor cocktails (Sigma, Cat#P8340) as described previously [50]. Samples were run on 4–20% TGX gels, transferred to nitrocellulose membrane, and probed with anti-COX7a2L (COX7RP) antibodies from Sigma. Hsp60 was used as loading control (Cell Signaling).

### RyR2 immunoprecipitation and immunoblotting

For immunoprecipitation of RyR2, cultured rat VCMs infected with Adv-COX7RP and Adv-shRNA COX7RP for 48 h were lysed using cell lysis buffer from Cell Signaling (catalogue no. 9803S), supplemented with phosphatase (Calbiochem; catalogue no. 524625) and protease inhibitor cocktails (Sigma; catalogue no. P8340). An overnight immunoprecipitation of RyR2 was performed at 4 °C using the Catch and Release v2.0 Kit (Millipore; catalogue no. 17‐500) in accordance with the manufacturer’s instructions using anti‐RyR2 antibody (Alomone catalogue no. ARR-002, 5 μg of antibody) and a negative control antibody comprising normal rabbit IgG (Millipore; catalogue no. 12-370, 5 μg of antibody). Samples were analyzed by immunoblotting.

To determine oxidation status of RyR2, the Oxidized Protein Western Blot Kit (Abcam catalogue no. ab178020) was used, whereby carbonyl groups of immunoprecipitated RyR2 were derivatized to 2,4 dinitrophenylhydrazone (DNP) by reaction with 2,4 dinitrophenylhydrazine [23]. For control, we used Derivatization Control Solution included in the kit. The DNP-RyR2 protein samples were separated on 4–20% Mini-PROTEAN TGX gels (Bio-Rad Laboratories, Cat#456-1094) and DNP-associated signal was assessed by the kit-provided anti-DNP rabbit primary antibody and anti-RyR2, followed by HRP-conjugated anti-rabbit goat lgG(H+L), secondary antibody.

Blots were developed with ECL (Bio-Rad Laboratories) and quantified using Image J (US National Institutes of Health, Bethesda, MD, USA) and Origin 8 software.

### Blue native PAGE

For the assessment of native protein complexes using blue native polyacrylamide gel electrophoresis (BN‐PAGE) [20], we used mitochondrial fractions isolated from LV heart tissues suspended in buffer containing 225 mmol/L mannitol, 70 mmol/L sucrose, 10 mmol/L HEPES, and 1 mmol/L EGTA (pH 7.4) [31]. The tissue was placed in a pre‐cooled 5 mL Wheaton™ Potter‐Elveheim Tissue Grinder (Fisher Scientific, Hampton, NH, USA; catalogue no. 22‐290067) and homogenized. Tissue homogenate was centrifuged at 700*g* for 10 min. The pellet consisting of nuclei and cell debris was discarded and the supernatant was then centrifuged at 20,000*g* for 15 min. The pellet was considered as mitochondrial enriched fraction. Samples were then solubilized using the mild detergent digitonin (1%) from NativePAGE™ Sample Prep Kit (Invitrogen; catalogue no. BN2008) in accordance with the manufacturer's instructions. To demonstrate changes in association of supercomplexes in VCMs infected with adenoviruses, cells were lysed in digitonin (1%) for 30 min on ice, and centrifuged at 20,000*g* for 30 min. Processed samples were resolved on NativePAGE™ 4–16% Bis‐Tris protein gels, 1.0 mm, 15‐well (Invitrogen; catalogue no. BN2011B10) in Native-Page cathode and anode buffers electrophoresed at 150 V for 1.5. followed by 250 V for 2 h. Samples were transferred onto nitrocellulose membranes before being probed with antibodies, as described above. The primary antibodies used were as follows: Complex I—anti-NDUFA9; Complex III—anti-UQCRFS1; Complex IV—anti-COX IV; all from Abcam. Hsp60 was used as loading control. Anti-Hsp60 antibodies were from Cell Signaling. The secondary antibodies used were goat anti‐mouse and goat anti‐rabbit ones (Promega, Madison, WI, USA). Blots were developed with ECL (Bio-Rad Laboratories) and quantified using Image J (US National Institutes of Health) and Origin 8 software.

### Oxygen consumption measurements

To assess mitochondrial function, oxygen consumption rates (OCR) of H9c2 cells infected with Adv-COX7RP-FLAG and Adv-shRNA COX7RP were measured by the Agilent Seahorse XFe96 extracellular flux analyzer (Seahorse Bioscience, North Billerica, MA). Briefly, cells were seeded in XFe96 cell culture plates at 10,000 cells/well in standard DMEM + 10% FBS medium and placed in a 37 °C incubator with 5% CO_2._ Adv-shRNA COX7RP was used for infection on day one and Adv-COX7RP on day 2. On day 3, cells were washed twice with Seahorse assay medium (XF base medium supplemented with 10 mmol/L glucose, 2 mmol/L glutamine, and 1 mmol/L sodium pyruvate; pH 7.4) and incubated in a 37 °C incubator without CO_2_ for 1 h. Oxygen consumption rate (OCR) was measured according to manufacturer’s guidelines of the XF Cell Mito Stress Test kit (Agilent). Oligomycin A (1 μmol/L final concentration), FCCP (1.5 μmol/L), and a combination of rotenone and antimycin A (1 μmol/L each) were injected on cells as indicated. After the assay, cells were stained with Hoechst dye and nuclei counted on a Cytation 5 cell imaging plate reader (Agilent) to normalize results. Data are expressed as pmol O_2_/min/100 cells. Plate location was randomized between groups in each assay. Wells with no response to oligomycin, FCCP, or rotenone/antimycin were removed from analysis based on the assumption of premature injection/leak of assay drugs into the well from upper plate reservoir, which confounds interpretation. There were no differences in the proportion of these wells between groups.

### Isolated heart oxygen consumption measurements

Male and female rats of equal age and approximately 300–500 g were heparinized, anesthetized with 3% isoflurane and heart and lungs removed en bloc following sternotomy. All procedures were approved by the Providence VAMC IACUC committee. Hearts were rapidly placed in ice-cold Krebs buffer, the aorta cannulated, and retrograde flow initiated with oxygenated Krebs buffer (in mmol/L: 118.00 NaCl, 4.70 KCl, 1.40 CaCl_2_, 1.70 MgSO_4_, 24.88 NaHCO_3_, 6.00 glucose, 1.20 KH_2_PO_4_, and 2.00 Na pyruvate) to wash blood and supply oxygen to the heart during working heart preparation. Pulmonary veins were ligated, the lungs removed under a microscope and the heart rapidly moved to working heart apparatus (IHS5, Hugo Sachs Elektronik, March, Germany). Hearts were then slowly increased to a constant perfusion pressure of 70 mmHg over 5 min. A cannula was inserted into the left atria and a pressure catheter inserted into the LV through the base of the heart with care to avoid large coronary vessels. A small catheter attached to an in-line optical O_2_ sensor (PreSens Precision Testing, Regensburg, Germany) and withdrawal syringe pump was placed in the pulmonary artery to continuously monitor O_2_ tension and temperature of coronary effluent. An additional in-line O_2_ sensor monitored O_2_ and temperature of perfusate. Hearts were switched to working mode via opening gravity flow of oxygenated Krebs buffer to the left atria (preload set to 10 mmHg). Langendorff perfusion was reduced and hearts allowed to equilibrate for 5 min before O_2_ consumption measurements. Coronary flow was assessed via eluent collection over 1 min. O_2_ consumption was normalized to heart weight and coronary flow and calculated according to Neely et al. [41].

### Glutathione GSH/GSSG measurement

Tissue total GSH and GSSG in rat left ventricles were measured using assay kit from Sigma-Aldrich (Cat MAK440) [34]. Briefly, stored tissue chunks were weighed and homogenates were prepared using Bullet Blender Strom 24 (Next Advance Inc, NY) in PBS, pH 7.4, 1 mmol/L EDTA with or without scavenger (provided with the kit) for the measurement of GSSG and total GSH respectively. All the samples were deproteinated with 5% meta-phosphoric acid. The clarified and diluted samples were mixed with working reagent and spectrophotometric reading was obtained at 412 nm at 0 and 10 min using SpectraMax 190 microplate reader (Molecular Devices, CA). Total GSH, reduced GSH, and GSSG were calculated following assay protocol.

### Data presentation and statistics

Group sample sizes were determined by power analysis based on our previous experience [20] performing two-tailed Student’s *t*-test to provide adequate power to detect 20% difference, assuming power of 80% (β = 0.80) and an α of 0.05.

No animals or samples were excluded from the study. Animals were grouped with no blinding, but randomized in cellular experiments. Data acquisition and analysis were not fully blinded as the same person carried out these processes.

Representative traces for cellular imaging experiments and representative western blot images were chosen to closely match the mean for the parameters assessed. Statistical analyses were performed using Origin 2020Pro (OriginLab) and R software [20, 47]. For each experiment, the number of animals (uppercase n [N]) and the number of VCMs (lowercase n) used is indicated. Data is expressed as mean ± SEM. *P* values are provided with two significant figures, where < 0.050 were considered significant (*). For experiments involving multiple VCMs (data points) isolated from one individual rat heart, a hierarchical level random intercept model was used [47]. The model tests for data clustering for each cell isolation and adjusts for any clustering with significance testing. Posthoc pairwise comparisons were carried out for hierarchical testing. No experiment-wide/across-test multiple test correction was applied; only within-test corrections were made. Statistical tests used for each dataset are presented in Table 3.

**Table 3:** Statistical tests used in this study

Figure	Type of sample	Statistical test applied
1B	One rat per N, multiple cells (n) used per rat	Two-level random intercept model with Tukey’s posthoc
1D	One rat per N, multiple cells (n) used per rat	Two-level random intercept model with Tukey’s posthoc
2D	One rat per N, multiple cells (n) used per rat	Two-level random intercept model with Tukey’s posthoc
3B-F	One rat per N, multiple cells (n) used per image per rat	Three-level random intercept model with Tukey’s posthoc
4B	LV tissue, one rat heart per N	Two-sample Student’s *t* test
4C	Ventricular tissue, one rat heart per N	Two-sample Student’s *t* test
5B	One rat per N, multiple cells (n) used per rat	Two-level random intercept model with Tukey’s posthoc
5D	One rat per N, multiple cells (n) used per rat	Two-level random intercept model with Tukey’s posthoc
6C	One rat per N, multiple cells (n) used per rat	Two-level random intercept model with Tukey’s posthoc
6E	LV tissue, one heart (rat/human) per N	Two-sample Student’s *t* test
7B	One rat per N, multiple cells (n) used per rat	Transient amplitude/decay/latency—two-level random intercept model with Tukey’s posthoc Ca^2+^ waves—Fisher’s exact test
7E	One rat per N, multiple cells (n) used per rat	One Way ANOVA with Tukey’s posthoc
8C	One rat heart per N	Two-sample Student’s *t* test
8D	One rat heart per N	Two-sample Student’s *t* test
8E	One rat heart per N	Two-sample Student’s *t* test
9A	LV tissue, one rat heart per N	Two-sample Student’s *t* test
9B	LV tissue, one human heart per N	Two-sample Student’s *t* test
9C	LV tissue, one rat heart per N	Two-sample Student’s *t* test
9D	LV tissue, one human heart per N	Two-sample Student’s *t* test
10B	LV tissue, one human heart per N	Two-sample Student’s *t* test
10D	LV tissue, one rat heart per N	Two-sample Student’s *t* test
10F	LV tissue, one rat heart per N	Two-sample Student’s *t* test
11C	H9c2 cells, one independent experiment per N	One way ANOVA with Tukey’s postdoc
12B	Isolated VMs from one rat heart per N	Two-sample Student’s *t* test
12D	Isolated VMs from one rat heart per N	Two-sample Student’s *t* test
13A	Isolated VMs from one rat heart per N	Two-sample Student’s *t* test
13B	Isolated VMs from one rat heart per N	Two-sample Student’s *t* test
13C	One rat per N, multiple cells (n) used per rat	Two-level random intercept model with Tukey’s posthoc
13D	One rat per N, multiple cells (n) used per rat	Two-level random intercept model with Tukey’s posthoc
14B	One rat per N, multiple cells (n) used per rat	Two-level random intercept model with Tukey’s posthoc
14E	Isolated VMs from one rat heart per N	Two-sample Student’s *t* test

## Results

### Increased spontaneous SR Ca^2+^ release in rat male VCMs vs females under β-adrenergic stimulation

**Fig. 1:**
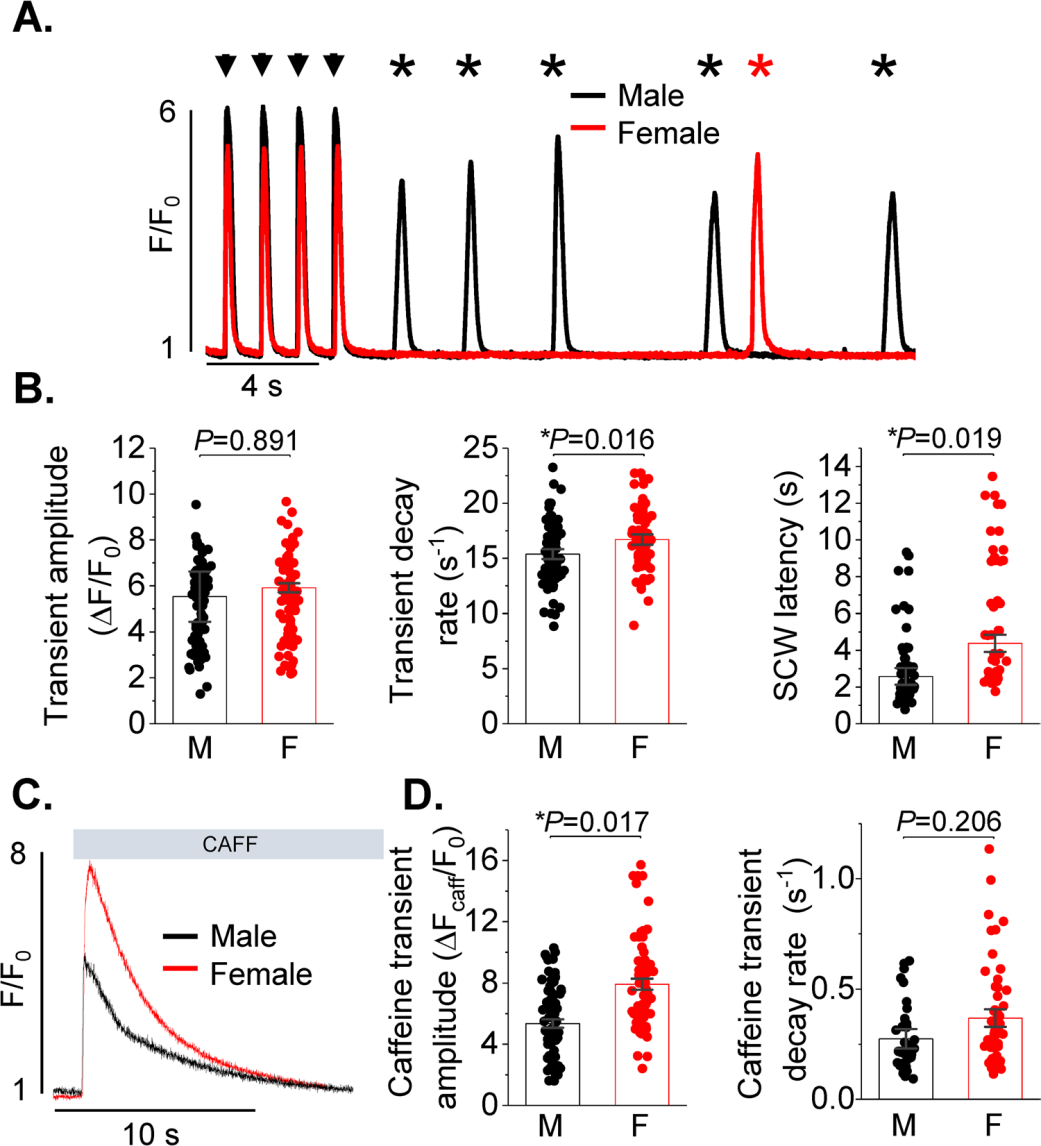
Intracellular Ca^2+^ handling is divergent in healthy male and female ventricular myocytes. **A** Fluo-3 fluorescence (F/F_0_) profiles of isoproterenol treated (ISO, 50 nmol/L) rat ventricular cardiomyocytes (VCMs) undergoing 1 Hz pace-pause protocol. Arrows depict field stimulation-evoked Ca^2+^ transients, stars show proarrhythmic diastolic Ca^2+^ waves. **B** Mean ± SEM Ca^2+^ transient amplitude (ΔF/F_0_) and decay rate (s^−1^), n = 90 male (M) and n = 90 female (F) VCMs; and spontaneous Ca^2+^ wave (SCW) latency (s), n = 62 M and n = 59 F VCMs. N = 11–12 M and N = 11–13 F animals. **C** Representative traces of caffeine-induced Ca^2+^ transients (10 mmol/L). **D** Mean ± SEM caffeine transient amplitude (ΔF/F_0_) and decay rate (s^−1^), n = 69 M, n = 75 F VCMs, N = 11 M, N = 13 F animals. *p < 0.05, *p* values were calculated using two-level random intercept model

Beta-adrenergic stimulation increases global pro-arrhythmic spontaneous Ca^2+^ release events, SCWs [9]. In order to assess potential differences in intracellular Ca^2+^ homeostasis, male and female VCMs were loaded with Ca^2+^ indicator Fluo-3AM and treated with 50 nmol/L β-adrenergic agonist isoproterenol (ISO). After 5 min incubation in ISO, VCMs were subjected to field stimulation at 1 Hz for 5 min at room temperature. Ca^2+^ transients and SCWs were recorded using a Leica SP8 confocal microscope in line scan mode. Representative cell-averaged fluorescence profile traces (male black, female red) are presented in Fig. 1A. Stimulated Ca^2+^ transients are indicated with arrows, while spontaneous global SR Ca^2+^ releases are denoted with asterisks. Analysis of Ca^2+^ transient amplitudes expressed as ΔF/F_0_, where F_0_ is basal fluorescence and ΔF = F − F_0_, revealed no difference between sexes (Fig. 1B left panel). Ca^2+^ transient decay in male VCMs showed small but significant slowing in comparison to females, indicative of reduced SERCA2a activity (Fig. 1B center panel). Furthermore, male VCMs exhibited shorter latency of SCW initiation vs females (Fig. 1B right panel). Latency, a well-established readout of RyR2-mediated SR Ca^2+^ release refractoriness [4, 11], was measured as time interval from the start of the last stimulated Ca^2+^ transient in the train to the initiation point of the first SCW. Total SR Ca^2+^ content was measured by rapid 10 mmol/L caffeine application at the end of the experiment (Fig. 1C). As shown in Fig. 1D, SR Ca^2+^ content was significantly higher in ISO-treated female rat VCMs in comparison to males. The decay rates of the caffeine transient were not different between male and female VCMs, indicative of unchanged Na^+^/Ca^2+^ (NCX1) exchanger activity. Taken together, these data suggest that SERCA2a activity is reduced, while the activity of the SR Ca^2+^ release channel RyR2 is significantly higher in male VCMs than in females under β-adrenergic stimulation.

### Decreased mito-ROS levels in female VCMs vs males under β-adrenergic stimulation

**Fig. 2:**
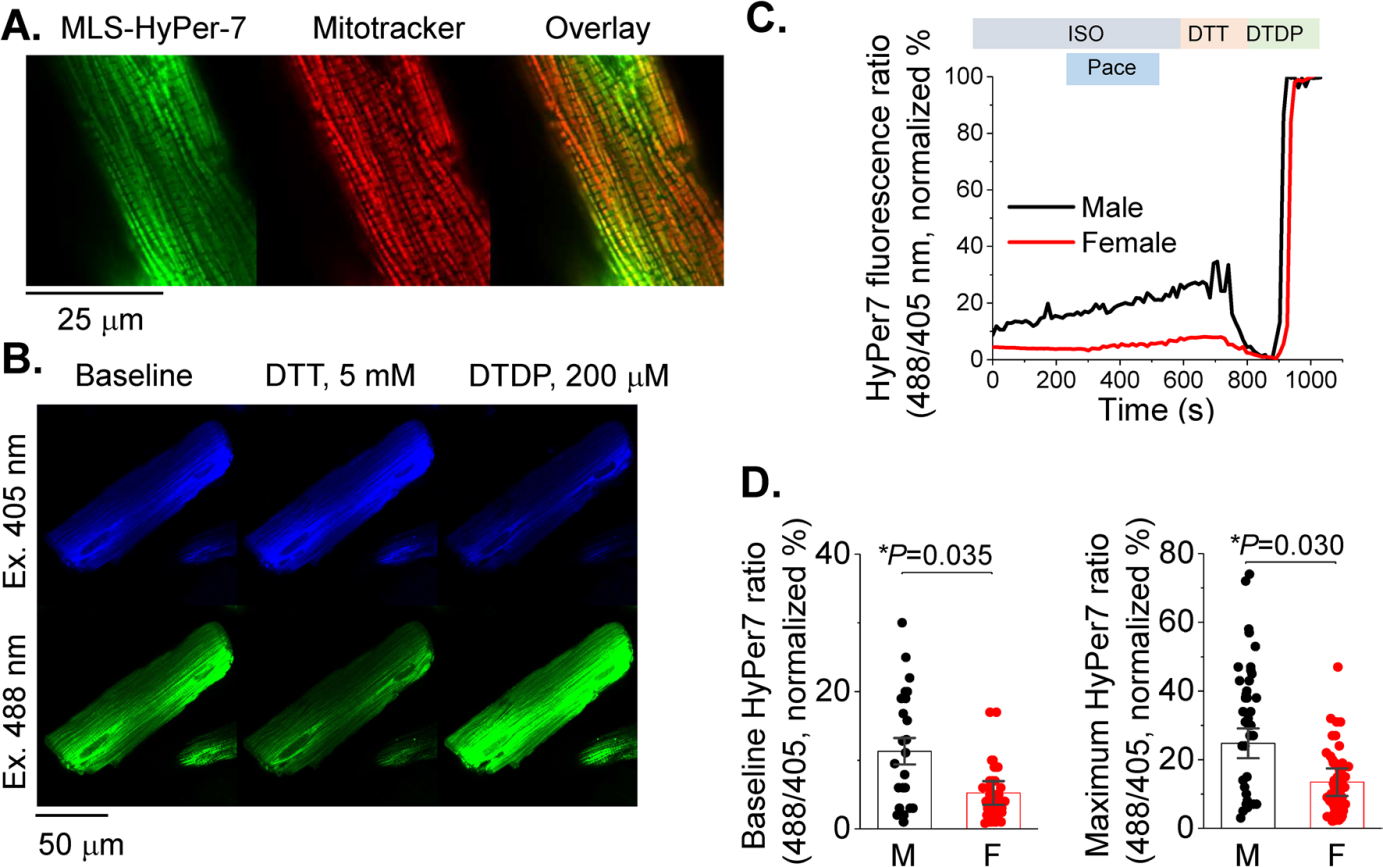
Matrix ROS biosensor MLS-HyPer7 reveals increased mito-ROS in male vs. female ventricular myocytes. **A** Mitochondrial MLS-Hyper-7 localization in live ventricular cardiomyocytes (VCMs) validated by Mitotracker. **B** Representative images of VCM infected with MLS-HyPer7, and treated with DTT (5 mmol/L) followed by DTDP (200 µmol/L) to achieve minimum and maximum fluorescence, demonstrating probe sensitivity. **C** Representative MLS-HyPer-7 recorded in male (M) and female (F) VCMs, respectively. Myocytes were treated with isoproterenol (ISO, 50 nmol/L) and paced at 1 Hz for 5 min. Fluorescence was normalized to minimum (DTT, 5 mmol/L) and maximum (DTDP, 200 µmol/L) fluorescence. **D** Mean ± SEM MLS-HyPer7 fluorescence, n = 37 M and n = 77 F VCMs, N = 11 M, N = 11 F animals. *p < 0.05, *p* values were calculated using two-level random intercept model

**Fig. 3:**
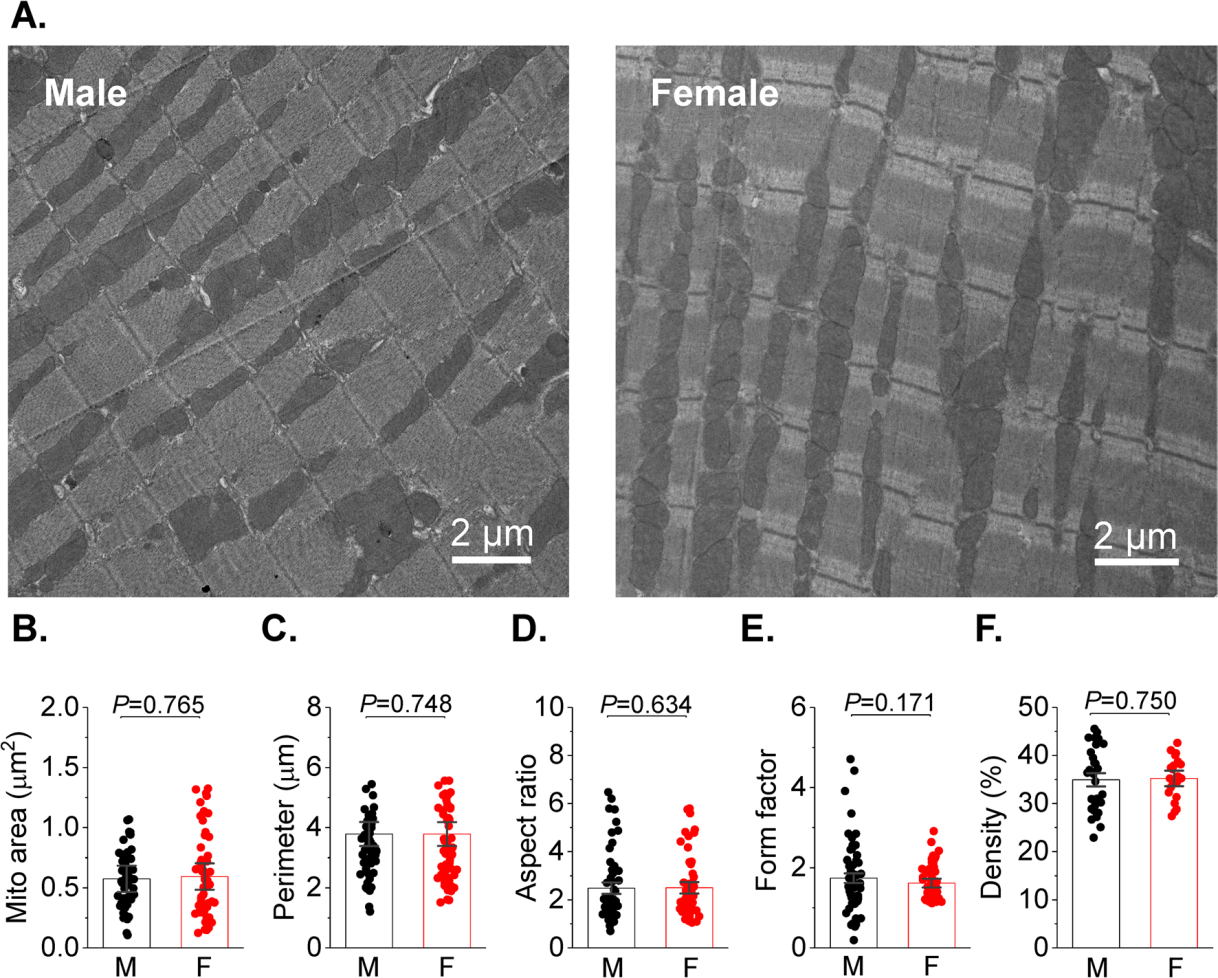
Absence of sex‐related differences in mitochondrial counts and morphology in rat heart. **A** Mitochondrial density assessed by transmission electron microscopy (TEM) in the left ventricular cardiac tissue from male (M) and female (F) rats. Original magnification is × 3200 and the white bars represent 2 μm scale. Heart were fixed with 2.5% glutaraldehyde solution then processed for transmission electron microscopy. **B**–**F** Mitochondrial morphometric parameters, including mito area (**B**), perimeter (**C**), aspect ratio (**D**), form factor and density (% of the cell area, **F**), in male (M) and female (F) rat hearts. The analysis was performed randomly from 60 M (30 images), 60 F (21 images) cardiac mitochondria from three animals per group and were analyzed by three-level random intercept model

**Fig. 4:**
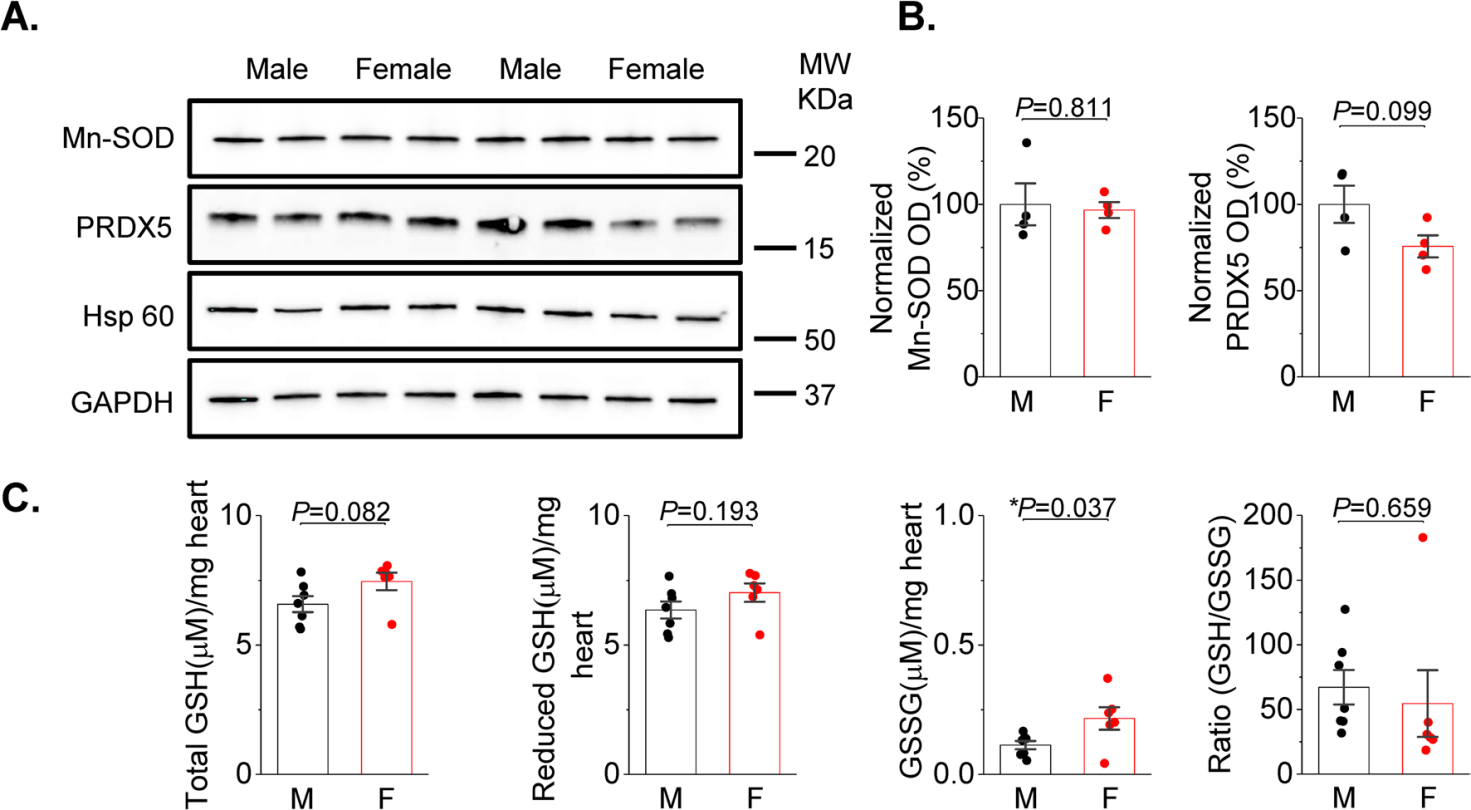
Anti-oxidant defenses in male and female left ventricular cardiac tissues. **A**, **B** Representative western blots and pooled data for mitochondrial superoxide dismutase Mn-SOD and peroxiredoxin 5 (PRDX5) normalized optical density from male (M) and female (F) rat left ventricular (LV) tissue samples. GAPDH and Hsp60 was used as loading control. Mean ± SEM, N = 4 M and N = 4 F rat LV samples; *p < 0.05, Student’s t-test. **C** Total tissue GSH, reduced GSH, oxidized GSH (GSSG), and the ratio of GSH/GSSG was measured from male and female rat ventricles. Mean + SEM, N = 7 M and N = 6 F rat, *p < 0.05, Student’s *t*-test

Increased activity of RyR2 and decreased SERCA2a activity may be explained by mito-ROS-mediated oxidation of these proteins [19]. To test this possibility, we expressed the dual excitation wavelength ROS sensor MLS-HyPer7 targeted to the mitochondrial matrix in VCMs [24]. Correct MLS-HyPer7 localization was confirmed by co-staining VCMs with mitochondria-specific dye Mitotracker Red (Fig. 2A). The MLS-HyPer7 signal was normalized to minimal fluorescence obtained by application of 5 mmol/L ROS scavenger DTT, and maximum fluorescence obtained by treatment of cells with 200 μmol/L DTDP, an oxidizing agent (Fig. 2B). Figure 2C demonstrates representative normalized traces of MLS-HyPer7 fluorescence ratio (male black, female red). After 5 min incubation in 50 nmol/L ISO, VCMs were field-stimulated for 5 min before application of DTT and DTDP. As shown in Fig. 2D, male ISO-treated VCMs exhibit a higher MLS-HyPer7 signal than female myocytes both before and during field stimulation. Of note, it was previously shown using TEM that mitochondria content and size differ in VCMs from male and female mouse hearts [30]. However, our TEM experiments in rat hearts showed no significant differences between sexes (Fig. 3). Therefore, sex differences in MLS-HyPer7 fluorescence cannot be ascribed to differences in mitochondria content in rat VCMs. In addition, as the probe is ratiometric as well as normalized to maxima and minima, there is minimal concern for sex-dependent expression differences. There is a possibility that heart antioxidant defenses differ between sexes as shown in other tissues [13]. To test this we performed western blot analysis of mitochondrial superoxide dismutase (MnSOD) and Peroxiredoxin-5 (PRXD5) and found no differences in samples from male and female rat cardiac tissues (Fig. 4A, B). Furthermore, we did not find statistically significant difference in GSH/GSSG ratio (Fig. 4C), indicating that there are no sex-dependent differences in antioxidant capacity. Taken together, these data are in line with previous reports that male VCMs exhibit significantly higher mito-ROS production than female VCMs [14, 51].

**Fig. 5:**
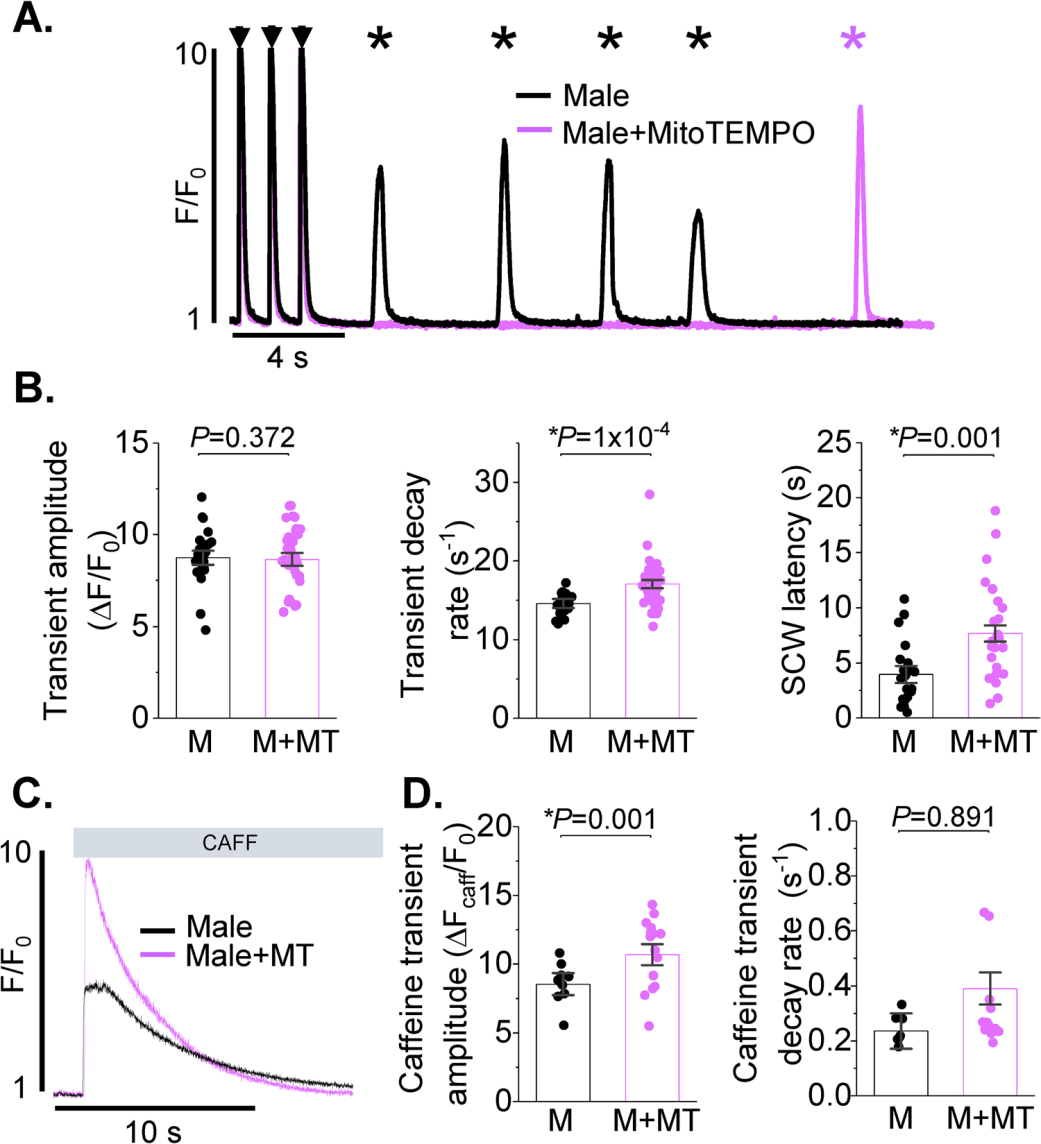
Mitochondrial ROS scavenging reduces spontaneous Ca^2+^ release is male ventricular myocytes. **A** Fluo-3 fluorescence (F/F_0_) profiles of isoproterenol treated (ISO, 50 nmol/L) rat ventricular cardiomyocytes (VCMs) undergoing 1 Hz pace-pause protocol. Arrows depict field stimulation-evoked Ca^2+^ transients, stars show proarrhythmic diastolic Ca^2+^ waves. Pretreatment with MitoTEMPO, a mitochondria-specific ROS scavenger (20 mmol/L, 8 min) reduces spontaneous Ca^2+^ waves. **B** Mean ± SEM Ca^2+^ transient amplitude (ΔF/F_0_) and decay rate (s^−1^), n = 26 male (M) and n = 37 MitoTEMPO treated male (M + MT) VCMs; and spontaneous Ca^2+^ wave (SCW) latency (s), n = 23 M and n = 25 M + MT VCMs. N = 4 M and N = 4 M + MT animals. **C** Representative traces of caffeine-induced Ca^2+^ transients (10 mmol/L). **D** Mean ± SEM caffeine transient amplitude (ΔF/F_0_) and decay rate (s^−1^), n = 11 M, n = 15 M + MT VCMs, N = 4 M, N = 4 M + MT animals. *p < 0.05, *p* values were calculated using two-level random intercept model

To test if increased mito-ROS plays a significant role in SR Ca^2+^ release destabilization we performed experiments treating male rat VCMs with MitoTEMPO, a mitochondria specific ROS scavenger (20 μmol/L, 20 min preincubation [24]). As depicted in Fig. 5, MitoTEMPO significantly attenuated SCWs, accelerated Ca^2+^ transient decay, and increased SR Ca^2+^ load in periodically paced male VCMs treated with ISO (50 nmol/L, 5 min). These results highlight the major role of differential mito-ROS production in sex dimorphism in intracellular Ca^2+^ homeostasis in VCMs under β-adrenergic stimulation.

### Decreased mito-Ca^2+^ levels in female VCMs vs males under β-adrenergic stimulation

**Fig. 6:**
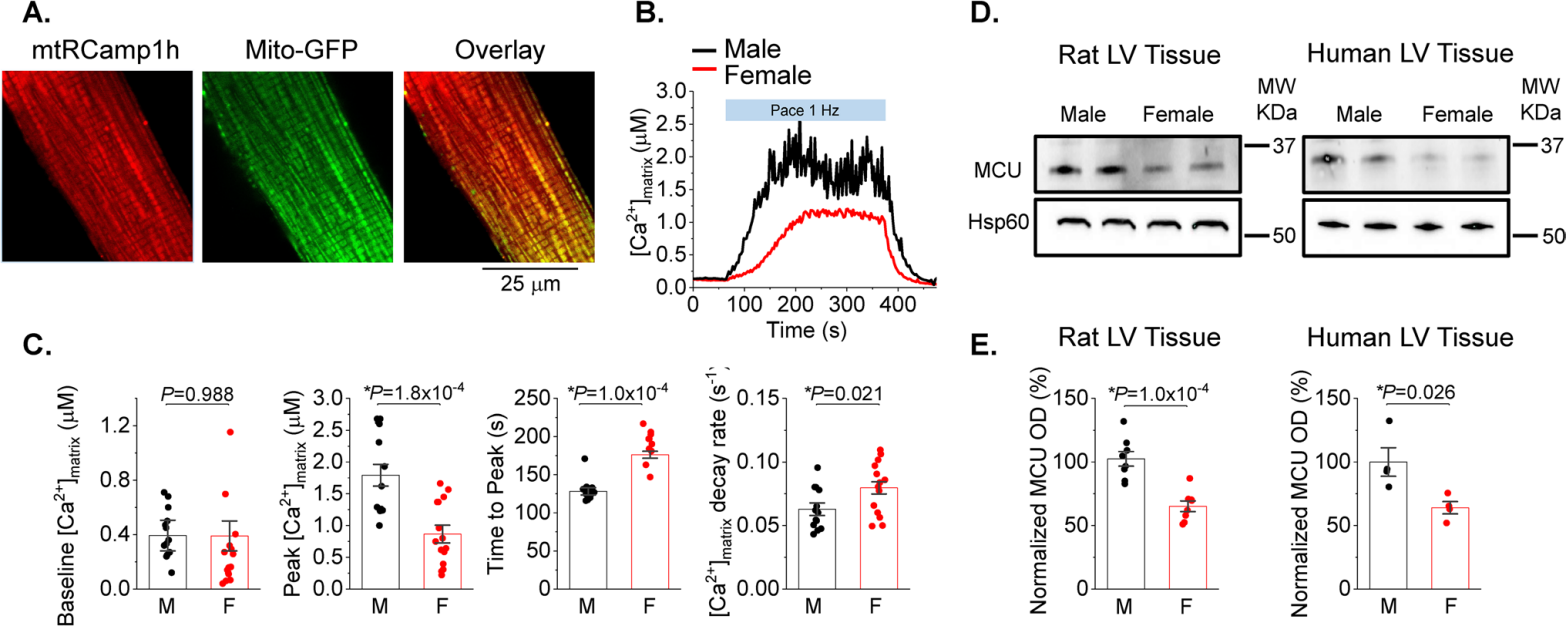
Female ventricular myocytes have reduced Ca^2+^ levels in mitochondria matrix vs. males. **A** Representative confocal images of a ventricular myocyte (VCM) infected with matrix-targeted mtRCamp1h (Kd ~ 1.3 mmol/L) and mito-GFP, with a merged figure demonstrating correct probe localization. **B** Representative time course of mtRCamp1h fluorescence recorded in male (M) and female (F) VCMs. Myocytes were treated with isoproterenol (ISO, 50 nmol/L) for 3 min before pacing at 1 Hz for 5 min. **C** Mean ± SEM baseline matrix [Ca^2+^], and peak matrix [Ca^2+^], time to peak matrix [Ca^2+^], and decay rate during pacing, n = 13 M and n = 15 F VMs. N = 8 M and 8 F animals. *p < 0.05, *p* values were calculated using two-level random intercept model. **D**, **E** Representative western blots and pooled data for COX7RP normalized optical density in rat and human left ventricular (LV) tissue samples. Mean ± SEM, N = 8 M and N = 8 F rat; and N = 4 M and N = 4 F human samples. *p < 0.05, Student’s *t*-test

We next tested potential differences between sexes in mito-[Ca^2+^], given that changes in matrix [Ca^2+^] are often associated with changes in mito-ROS production [24, 36, 48]. Using the matrix-targeted Ca^2+^ probe mtRCamp1h we determined that mitochondrial [Ca^2+^] is significantly lower in field-stimulated female VCMs pretreated with 50 nmol/L ISO for 5 min (Fig. 6A–C). Correct mitochondrial localization of mtRCamp1h was confirmed in VCMs co-expressing mito-targeted GFP. As seen in Fig. 6A, red mtRCamp1h fluorescent signal overlaps with green signal of mito-GFP. Figure 6B shows representative mito-Ca^2+^ traces from male (black) and female (red) VCMs derived from mRCamp1h signal. To convert mtRCamp1h fluorescence into [Ca^2+^], cells were permeabilized with saponin at the end of recording and exposed to 2 mmol/L Ca^2+^ buffer EGTA to obtain Fmin followed by application 20 μmol/L [Ca^2+^] to obtain Fmax. Although no significant changes in mito-[Ca^2+^] were detected at rest, field stimulation of VCMs at 1 Hz revealed that in female VCMs it takes significantly longer to reach maximum mito-[Ca^2+^], and that this maximum concentration is significantly lower than in male VCMs (Fig. 6C). Importantly, western blot analysis revealed significantly lower expression levels of MCU in rat female VCMs vs males, which could potentially explain why matrix Ca^2+^ is lower (Fig. 6D). Notably, lower MCU levels were detected in female human LV donor heart (≤ 52 year old) samples vs males as well (Fig. 6E). Given that mito-[Ca^2+^], in addition to uptake, is determined by removal via mitochondrial Na^+^/Ca^2+^ exchanger [17], we assessed the rate of removal by measuring mito-Ca^2+^ decay upon cessation of pacing. Of note, the rate of Ca^2+^ removal from mitochondrial matrix was significantly faster in females.

### Aging abolishes sex differences in intracellular Ca^2+^ homeostasis and mito-ROS production

**Fig. 7:**
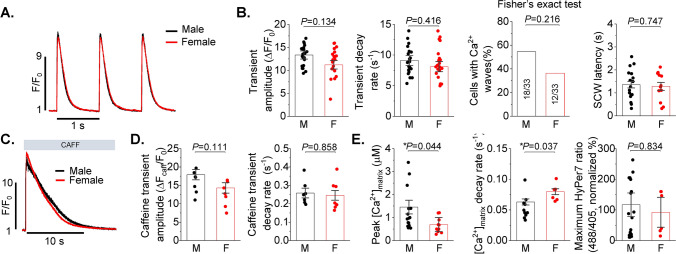
Intracellular Ca^2+^ handling is similar in male and female ventricular myocytes from aged (22 month old) hearts. **A** Fluo-4 fluorescence (F/F_0_) profiles of isoproterenol treated (ISO, 50 nmol/L) rat ventricular myocytes (VCMs) paced at 1 Hz. **B** Mean ± SEM Ca^2+^ transient amplitude (ΔF/F_0_) and decay rate (s^−1^), n = 23 male (M) and n = 25 female (F) VCMs; proportion of cells displaying Ca^2+^ waves following 2 Hz pacing and distribution of latency to Ca^2+^ waves (s), n = 33 for M and n = 33 for F, N = 4 M and F animals. **C** Representative traces of caffeine-induced Ca^2+^ transients. **D** Mean ± SEM caffeine transient amplitude (ΔF/F_0_) and decay rate (s^−1^), n = 7 M and F VMs, N = 4 M and F animals. *p* values were calculated using two-level random intercept model except where indicated. **E** Aged male (M) and female (F) VCMs exhibit similar mito-ROS levels, while sexual dimorphism in matrix [Ca^2+^] preserved with aging. VCMs were passed at 1 Hz for 5 min in the presence of 50 nmol/L ISO, Mean ± SEM, N = 2–4 M, 2–4 F, n = 6–20, *p < 0.05, *p* values were calculated using one way ANOVA

Using young and old VCMs from female rabbit hearts we previously showed that disturbances in Ca^2+^ handling are governed by increased mito-ROS production [15]. Here we tested potential sex differences in Ca^2+^ cycling in VCMs from 22-month-old F344 rats obtained from NIH aged rat colony. As demonstrated in Fig. 7, we find no differences in Ca^2+^ transient amplitude and decay rate, as well as SR Ca^2+^ content and propensity to generate pro-arrhythmic SCWs (Fig. 7A–D). This was accompanied by similar rates of mito-ROS production in old male and female VCMs (Fig. 7E), which is in striking contrast to young cells (Fig. 6B). Of note, the sex differences in mito-Ca^2+^ uptake and removal were preserved with aging (Fig. 7E), suggesting that the main source of age-related mito-ROS increase in females is not related to mito-Ca^2+^ mishandling.

### Female mitochondria exhibit higher levels of ETC supercomplexes and COX7RP

**Fig. 8:**
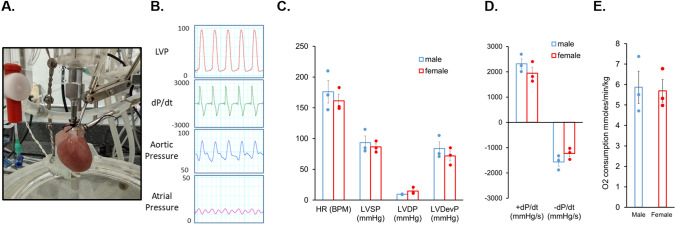
Oxygen consumption is similar in male and female working heart preparations. **A** Picture of rat working heart preparation. **B** Representative left ventricular (LVP), aortic and atrial pressure traces (mmHg) and LVP dP/dt of isolated rat heart in working mode. **C**–**E** Quantitation of male and female isolated rat heart myocardial O_2_ consumption N = 3 males and N = 3 females. *p* values were calculated using Student’s *t*-test

**Fig. 9:**
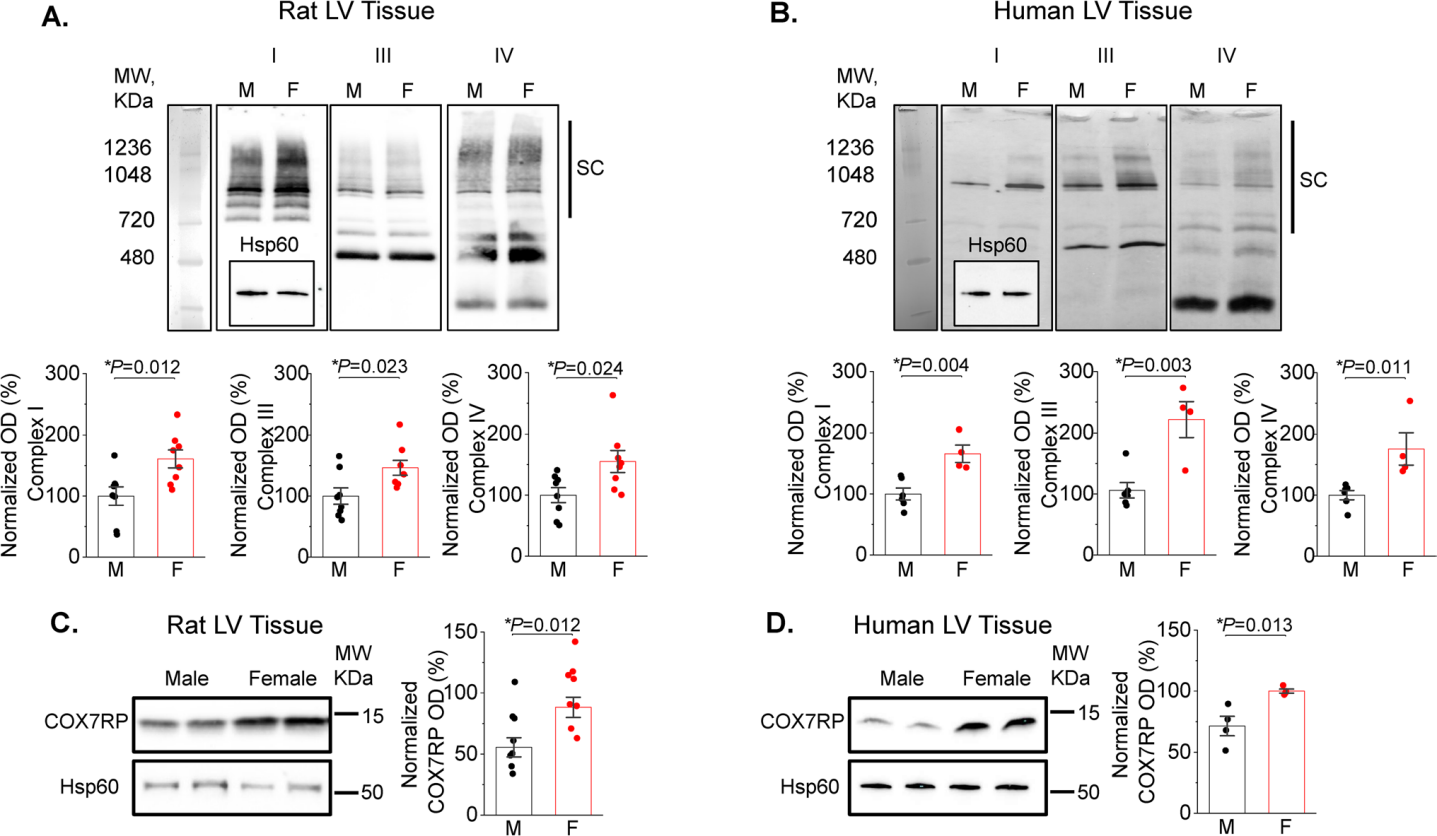
Female left ventricular tissues demonstrate increased mitochondrial supercomplex formation and expression of COX7RP compared to males. **A**, **B** Representative BN-PAGE images of ETC complexes from male (M) and female (F) rat (**A**) and human (**B**) left ventricular (LV) tissue samples. Antibodies used: Complex I—anti-NDUFA9; Complex III—anti-UQCRFS1; Complex IV—anti-COX IV all from Abcam. Hsp60 was used as loading control (inset, Anti-Hsp60 from Cell Signaling). Supercomplexes (SCs) are indicated by black bars. Mean ± SEM, N = 8 M and N = 8 F rat LV samples; and N = 6 M and N = 4 F human LV samples, *p < 0.05, Student’s t-test. **C**, **D** Representative western blots and pooled data for COX7RP normalized optical density in rat (**C**) and human (**D**) LV tissue samples. Hsp60 was used as loading control (inset, Anti-Hsp60 from Cell Signaling). Mean ± SEM, N = 8 M and N = 8 F rat samples; and N = 4 M and N = 4 F human samples, *p < 0.05, Student’s *t*-test

**Fig. 10:**
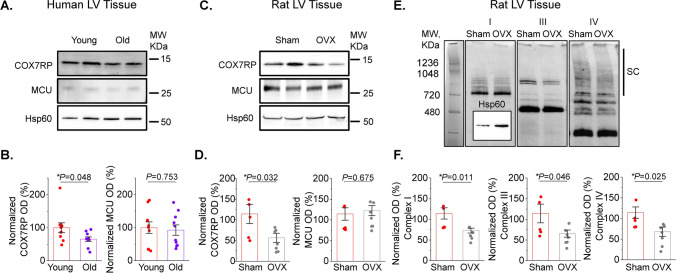
Reduced COX7RP levels in LV heart tissues from old female donors and ovariectomized female rats. **A**, **B** Representative western blots and pooled data for COX7RP and MCU normalized optical density in left ventricular (LV) tissue samples from young (less than 35 year old, N = 10) and old (more than 60 years old, N = 10) female donor hearts. Mean ± SEM, *p < 0.05, Student’s *t*-test. **C**, **D** Representative western blots and pooled data for COX7RP and MCU normalized optical density in left ventricular (LV) tissue samples Sham and ovariectomised (OVX) rats. Hsp60 was used as loading control (Cell Signaling). **E** Representative BN-PAGE images of ETC complexes from rat LV tissue samples. Antibodies used: Complex I—anti-NDUFA9; Complex III—anti-UQCRFS1; Complex IV—anti-COX IV; all from Abcam. Hsp60 was used as loading control (inset, Anti-Hsp60 from Cell Signaling). Supercomplexes (SCs) are indicated by black bar. **F** Pooled normalized optical density data for (**E**). Mean ± SEM, N = 5 Sham and N = 7 OVX, *p < 0.05, Student’s *t*-test

Differences in cardiac respiratory capacity between sexes has not been previously described [29, 30]. In line with this, our measurements of oxygen consumption in Langendorff-perfused working heart preparations showed no difference in myocardial O_2_ consumption between males and females (Fig. 8). Therefore, we surmised that a compensatory mechanism must exist to offset the effects of lower matrix [Ca^2+^] in females vs males. One plausible mechanism to explain this discrepancy could include enhanced incorporation of individual ETC complexes into ETC supercomplexes to increase electron transport efficiency [3, 12]. Indeed, as depicted in Fig. 9A, Blue Native Gel Electrophoresis (BN-PAGE) of isolated mitochondrial proteins showed increased high molecular weight assemblies consisting of comigrating subunits for Complex I, III and IV in rat female vs male LV tissues. Importantly, experiments using LV tissues from healthy human donor hearts exhibited a similar pattern (Fig. 9B). Notably, the risk of CVD and cardiac arrhythmia dramatically increases in postmenopausal females matching or even outpacing males [52], suggesting that hormonal changes may play a role in these processes. Of note, expression of COX7RP, a protein critically involved in supercomplex formation, is estrogen dependent [33, 53]. We tested potential sex differences in COX7RP expression levels and found that they are significantly higher in females than males both in rat and human hearts from heathy donors (Fig. 9C, D). Furthermore, using samples from young and old female human hearts we confirmed that COX7RP levels decrease with aging in contrast to MCU (Fig. 10A, B). Experiments using sham and ovariectomized (OVX) female rats showed an identical expression pattern (Fig. 10C, D). In addition to a decrease in COX7RP levels, we found that supercomplexes are significantly reduced in OVX LV tissue samples as well (Fig. 10E, F). These data support the key role of estrogen dependent COX7RP expression in supercomplex assembly.

### COX7RP regulates mitochondria oxygen consumption and supercomplexes

**Fig. 11:**
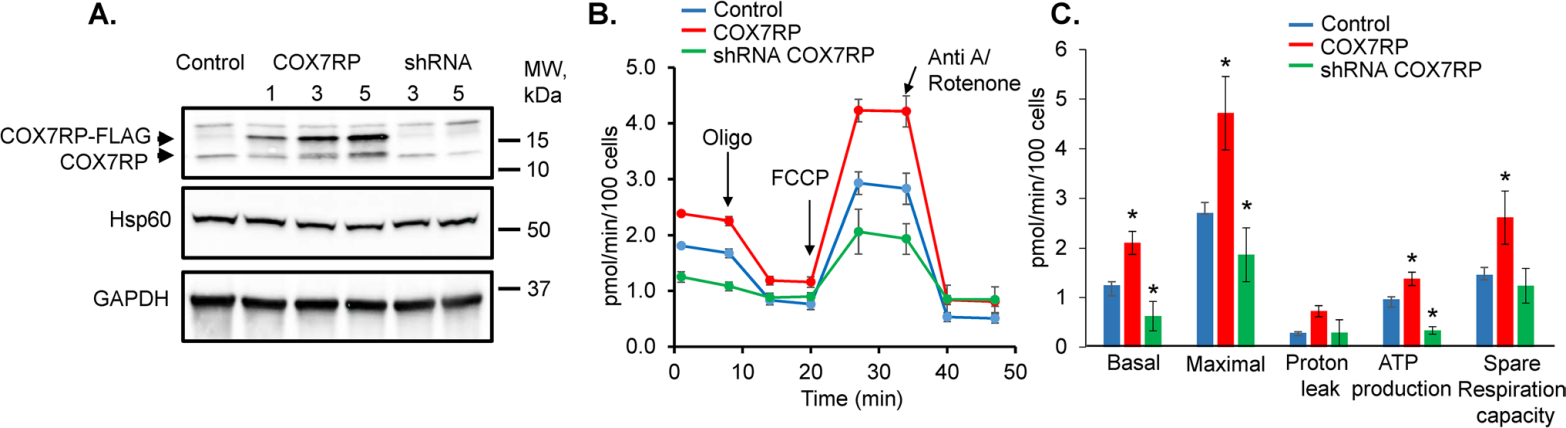
COX7RP modulates oxygen consumption in H9C2 myoblasts. **A** Representative western blot demonstrating efficiency of COX7RP overexpression and COX7RP shRNA constructs in H9C2 cells infected with adenoviruses for 48 h. Hsp60 and GAPDH were used as loading controls. **B** Representative Seahorse Analyzer recordings of oxygen consumption in H9C2 cells in control cells and cells expressing COX7RP or COX7RP shRNA. **C** Pooled respirometry data, Mean ± SEM, n ≥ 18, N = 3 independent experiments, *p < 0.05, one way ANOVA

**Fig. 12:**
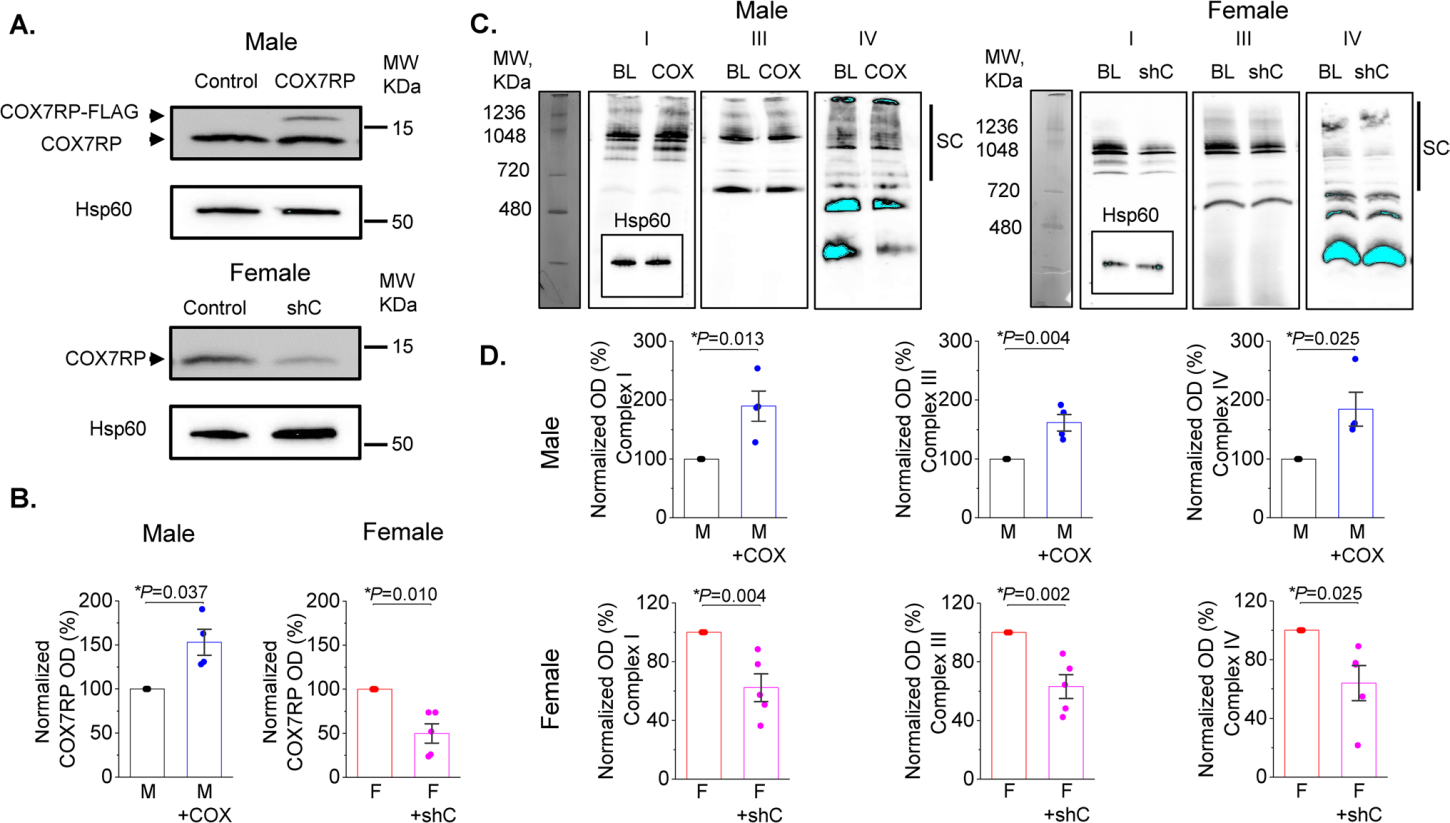
COX7RP expression levels regulate formation of mitochondrial supercomplexes in rat ventricular myocytes. **A**, **B** Transfection of rat ventricular cardiomyocytes with Ad-COX7RP-FLAG (male (M)) and with shRNA COX7RP (female (F)) alters expression levels of COX7RP protein 48 h after infection (10 MOI). Hsp60 was used as loading control. **B** Pooled data for (**A**), Mean ± SEM, N = 4 M and N = 5 F, *p < 0.05, Student’s *t*-test. **C** Representative BN-page experiments demonstrating that COX7RP overexpression increases formation of multimolecular ETC supercomplexes in Ms, while COX7RP shRNA decreases it in Fs. Antibodies used: Complex I—anti-NDUFA9; Complex III—anti-UQCRFS1; Complex IV—anti-COX IV; anti-GAPDH all from Abcam. Hsp60 was used as loading control (inset, Anti-Hsp60 from Cell Signaling). Supercomplexes (SCs) are indicated by black bars. **D** Pooled normalized optical density data for (**C**) mean ± SEM, N = 4 M and N = 5 F animals, *p < 0.05, Student’s *t*-test

**Fig. 13:**
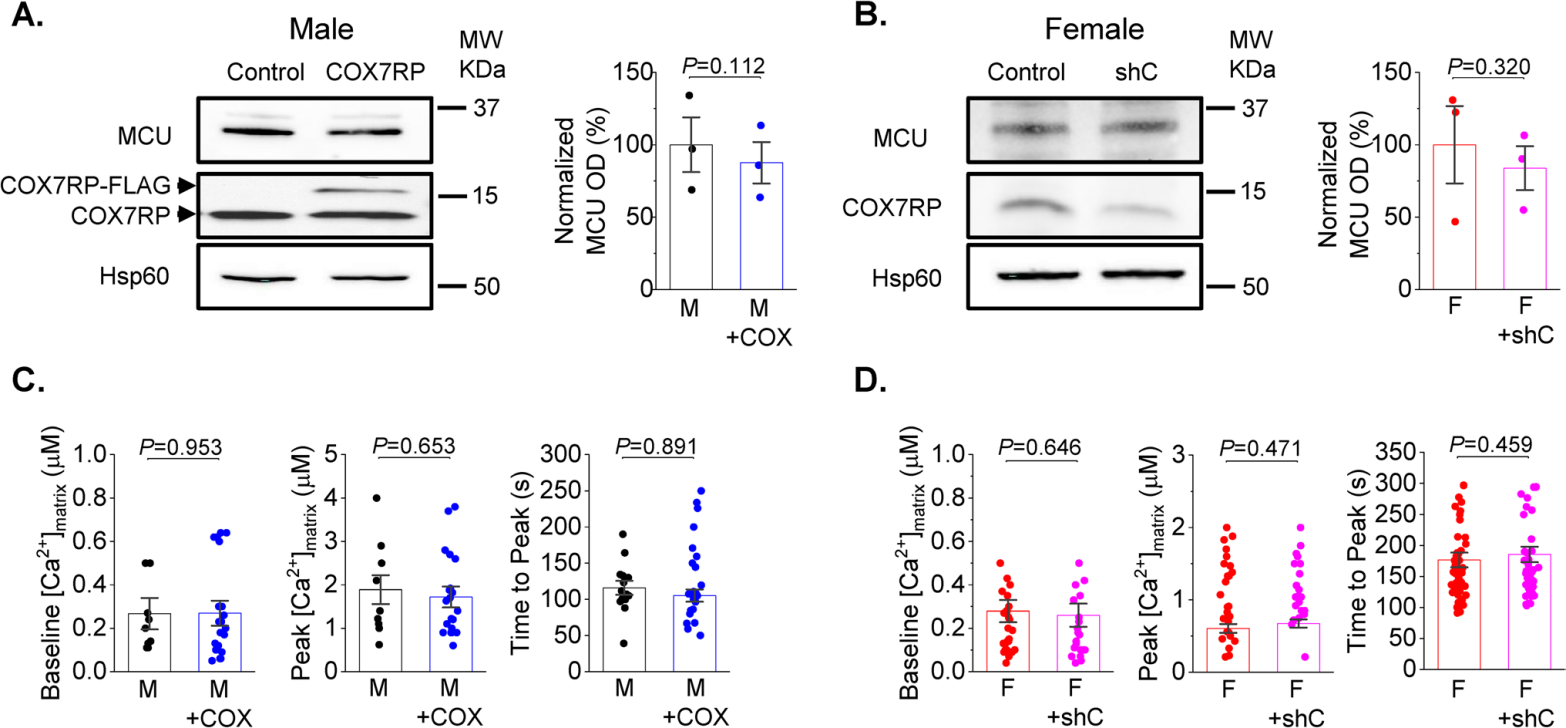
Altering COX7RP expression levels does not affect MCU expression and mito-Ca^2+^ uptake in male and female rat ventricular myocytes. **A**, **B** Transfection of rat ventricular cardiomyocytes (VCMs) with Ad-COX7RP-FLAG (male (M)) and with shRNA COX7RP (female (F)) does not change expression levels of MCU 48 h after infection (10 MOI). Hsp60 was used as loading control. Mean ± SEM, N = 4 M and N = 3 F, *p < 0.05, Student’s *t*-test. **C**, **D** Adenovirus-mediated changes in COX7RP expression levels do not affect mitochondrial Ca^2+^ uptake measured using mtRCamp1h Ca^2+^ biosensor in M and F VCMs. Mean ± SEM, n = 9 M and n = 20 M + COX, N = 4 M; n = 27 F and n = 21 F + shC and N = 3 F, *p < 0.05, *p* values were calculated using two-level random intercept model

In order to modify COX7RP expression levels, we utilized adenoviral vectors carrying human COX7RP-FLAG and shRNA COX7RP sequences. Efficiency of adenovirus-mediated COX7RP overexpression and shRNA-COX7RP knockdown (KD) was confirmed in H9c2 cells 48 h after transduction with western blot (Fig. 11A). As seen in representative western blot, FLAG-tagged COX7RP runs slower on the gel than wild type COX7RP. Mitochondrial protein Hsp60 was used as loading control. Next, we performed measurement of oxygen consumption in H9c2 cells with a Seahorse Analyzer in cells expressing shRNA-COX7RP or COX7RP. Averaged traces are presented in Fig. 11B. As seen in Fig. 11C, COX7RP overexpression increases basal, maximum and ATP-linked respiration in H9c2 cells, while COX7RP KD reduces it. Next, viral constructs were tested in cultured rat VCMs. COX7RP levels were increased ~ 50% in male VCMs 48 h after transduction (Fig. 12A, B). Expression of shRNA COX7RP (shC) led to ~ twofold reduction of the protein in female VCMs 48 h after infection (Fig. 12A, B). Next, samples from cultured male VCMs expressing COX7RP-FLAG and female VCMs expressing shRNA COX7RP were subjected to BN-PAGE analysis to test potential COX7RP influence on ETC supercomplex assembly (Fig. 12C, D). Importantly, COX7RP KD significantly reduced supercomplexes in female VCMs, while COX7RP overexpression increased them in VCMs from males. These data further corroborates the key role of COX7RP in mitochondrial ETC supercomplex assembly in cardiomyocytes. Importantly, altering COX7RP levels did not change MCU expression levels, mito-Ca^2+^ uptake and peak [Ca^2+^] in male and female rat VCMs (Fig. 13).

### COX7RP regulates SR Ca^2+^ release, mito-ROS production and RyR2 oxidation in VCMs

**Fig. 14:**
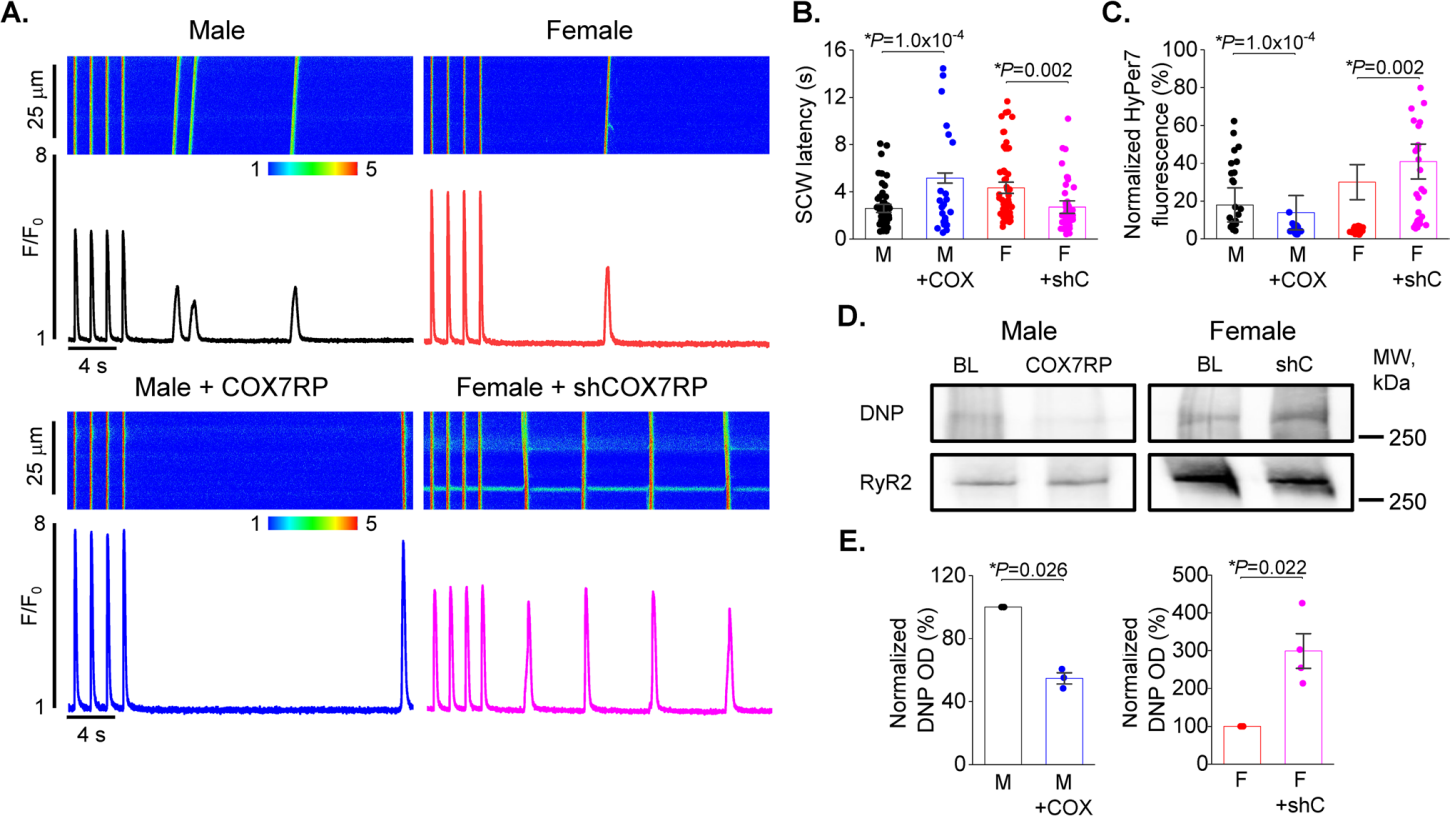
COX7RP overexpression reduces spontaneous SR Ca^2+^ release and mito-ROS in male ventricular myocytes under β-adrenergic stimulation. Conversely, COX7RP knock-down increases Ca^2+^ waves and mito-ROS in females. **A**, **B** Representative Ca^2+^ traces (**A**) and pooled data (**B**) for spontaneous Ca^2+^ waves (SCW) latency in male (M), female (F), male overexpressing COX7RP (M + COX), and female expressing shRNA COX7RP (F + shC) ventricular cardiomyocytes (VCMs) exposed to 50 nmol/L isoproterenol (ISO, 5 min) after cessation of field stimulation (1 Hz). Mean ± SEM, n = 58–64, N = 6–9 preparations, *p < 0.05, p values were calculated using two-level random intercept model. **C** Pooled data for normalized fluorescence of mitochondria matrix ROS biosensor MLS-HyPer-7. Mean ± SEM, n = 29–42 VCMs, N = 6–9 animals, *p < 0.05, *p* values were calculated using two-level random intercept model. **D** Adenovirus–mediated expression of COX7RP in males and shRNA COX7RP in females alter RyR2 oxidation levels in rat VMs exposed to isoproterenol (ISO, 50 nmol/L) paced at 1 Hz for 5 min cultured for 48 h after infection (10 MOI). Immunoprecipitated RyR2s from M and F cultured VCM samples were probed with Anti-DNP antibody to detect amount of oxidized cysteines. **E** Pooled DNP optical density normalized to corresponding RyR2 signal. Mean ± SEM, N = 3 M, N = 4 F. *p < 0.05, Student’s *t*-test

Next, we tested the effects of adenoviral-mediated COX7RP-FLAG overexpression in male VCMs and shRNA-mediated KD in females treated with ISO (50 nmol/L, 5 min) on RyR2-mediated spontaneous SR Ca^2+^ release. Representative line scan images and corresponding Fluo-3 fluorescence time profiles are depicted in Fig. 14A. The latency of SCWs appearing after cessation of 1 Hz field-stimulation was significantly lengthened in male VCMs overexpressing COX7RP, while COX7RP KD significantly shortened it (Fig. 14A, B). Importantly, parallel experiments in VCMs coexpressing mito-ROS sensor MLS-HyPer7 subjected to the same experimental protocol showed significant mito-ROS reduction in male COX7RP-overexpressing VCMs, while mito-ROS in female shRNA-COX7RP-expressing VCMs was significantly increased (Fig. 14C). We then assessed possible COX7RP expression-dependent changes in RyR2 oxidation state. Control and COX7RP-overexpressing male and shRNA COX7RP-expressing female VCMs were exposed to 50 nmol/L ISO pretreatment and then paced at 1 Hz for 5 min in the presence of ISO. Then cells were immediately collected for RyR2 immunoprecipitation. The relative changes in immunoprecipitated RyR2s oxidized cysteine content were assessed using anti-DNP antibodies [23]. As shown in Fig. 14D, COX7RP overexpression significantly reduces RyR2 oxidation in male VCMs, while COX7RP KD in female VCMs increases it. To summarize, COX7RP KD increased RyR2 oxidation, mito-ROS, and profoundly disturbed SR Ca^2+^ release in female VCMs promoting generation of pro-arrhythmic SCWs, while COX7RP overexpression led to opposite results in males.

## Discussion

In the present study we investigated the mechanisms underlying reduced mito-ROS production in female VCMs. We have shown that male rat VCMs exhibit higher propensity to pro-arrhythmic diastolic SR Ca^2+^ release governed by hyperactive RyR2s due to oxidation by mito-ROS. We found that lower mito-ROS production in females is accompanied by lower mito-[Ca^2+^] and heightened ETC supercomplex assembly. We found the more efficient electron transport in females is likely related to increased expression levels of the estrogen-dependent mitochondrial assembly protein COX7RP.

### Increased mito-ROS and unstable intracellular RyR2-mediated Ca^2+^ cycling in male vs female VCMs during β-adrenergic stimulation

Recent studies using mouse models with loss of MCU function revealed that SR Ca^2+^ release is directly coupled to mitochondrial metabolic output in stress conditions [38, 45]. β-adrenergic stimulation increases SR Ca^2+^ release amplitude to levels sufficient for MCU to transmit Ca^2+^ into the mitochondrial matrix [23], activating Ca^2+^-dependent dehydrogenases in the Krebs Cycle and promoting ATP production [21]. Thus, our main focus was to characterize potential differences in Ca^2+^ homeostasis in VCMs from female and male rat hearts in the presence of ISO (Fig. 1). Notably, although we did not find statistically significant differences in Ca^2+^ transient amplitudes in periodically stimulated male and female VCMs, we saw a significantly lower SR Ca^2+^ content in male VCMs. Furthermore, male VCMs exhibited shorter latency of SCW initiation. Together, these data are a strong indication that RyR2 activity is higher in male than in female VCMs under β-adrenergic stimulation. Previous reports directly linked impaired SR Ca^2+^ handling in VCMs incubated with β-adrenergic agonists to increased RyR2 oxidation by mito-ROS resulting in increased channel activity and high propensity to pro-arrhythmic SCWs [9, 15, 23]. In line with these findings, our measurements using the mitochondria-targeted ROS biosensor MLS-HyPer-7 suggest that sex differences in RyR2-mediated spontaneous SR Ca^2+^ release are indeed associated with different levels of mito-ROS (Fig. 2). Importantly, RyR2 hyperoxidation has been associated with reduced cardiac function and/or increased arrhythmic potential in various cardiac diseases including heart failure [49], infarct [5, 10], diabetic cardiomyopathy [7, 28, 37], age-related cardiac dysfunction [15, 18], and even inherited cardiac arrhythmia syndromes [23]. Increased mito-ROS production in VCMs from male hearts is expected to carry additional risk and may explain heightened protection in healthy premenopausal females.

Interestingly, the Ca^2+^ transient decay in ISO-treated female VCMs was ~ 10% faster than males (Fig. 1A, B). Changes in Ca^2+^ transient decay rate could be primarily ascribed to differences in SERCA2a expression and activity [6]. It is not likely that there are differences in SERCA2a expression levels between sexes [43]. However, SERCA2a activity can be modulated, i.e. lowered, by redox modification at cysteine 674 [44]. Slow Ca^2+^ transient decay in male VCMs treated with ISO is consistent with reduced SERCA2a activity under higher ROS levels. Importantly, treatment of male VCMs with MitoTEMPO significantly attenuated spontaneous SR Ca^2+^ release, increased SR Ca^2+^ load, and accelerated Ca^2+^ transient decay (Fig. 5). Taken together, our data suggest that high mito-ROS in ISO-treated male VCMs markedly disturbs intracellular Ca^2+^ homeostasis consistent with increased RyR2 activity and lower SERCA2a function.

### Sexual dimorphism in mitochondria [Ca^2+^] uptake and ETC supercomplex assembly in the heart

Mitochondria are increasingly acknowledged as a key target for sex differences in pathologies [51]. However, understanding of exact mechanisms underlying sex differences in mito-ROS production in health remains limited. Given that ETC is the major source of ROS in mitochondria, increased ETC activity under conditions of higher workload or stress is expected to accelerate ROS production [21, 26]. Indeed, we recently reported that MCU-mediated influx of Ca^2+^ into the mitochondrial matrix, which increases ETC activity by stimulating production of NADH and FAD_2_, is essential for mito-ROS production in VCMs challenged with β-adrenergic agonist [23]. Importantly, mito-[Ca^2+^] in VCMs rapidly changes in response to changes in frequency and amplitude of cytosolic Ca^2+^ transients, directly linking contractile activity and mitochondria ATP production to closely match varying metabolic demand with output. Our experiments in VCMs expressing mito-[Ca^2+^] biosensor mRCamp1h (Fig. 6A) showed that mito-[Ca^2+^] is drastically lower in field-stimulated ISO-treated VCMs from female rat hearts vs males (Fig. 6B, C), despite similar Ca^2+^ transient amplitudes (Fig. 1A, B). Previously it was reported that Ca^2+^ uptake is lower in isolated female rat cardiac mitochondria than in males [2]. The authors suggested that this decrease could be ascribed to lower MCU activity. In support of this notion, our western blot analysis clearly shows lower expression levels of MCU in mitochondria from female rat and human LV tissues vs males (Fig. 6D, E). Furthermore, our data suggests that Ca^2+^ removal from the matrix is more efficient in female mitochondria. Taken together, these results suggest that female cardiac mitochondria are less reliant on [Ca^2+^] accumulation to satisfy increased metabolic demand during high workload or stress.

Given that cardiac mitochondrial respiratory capacity is not known to be grossly different between sexes [29, 30] and cardiac output is similar even at higher workloads [25, 27], we surmised that a compensatory mechanism must exist to offset the effects of lower matrix [Ca^2+^] in females vs males. We confirmed that despite lower mitochondrial [Ca^2+^] in female VCMs, female working hearts have the same myocardial O_2_ consumption as male hearts when operating at similar work levels (Fig. 8). Mitochondrial supercomplex assembly is one plausible mechanism of respiratory compensation in females [14]. Furthermore, more compact ETC organization is expected to reduce emission of ROS due to more efficient electron transport. Indeed, our BN-PAGE experiments demonstrate higher abundance of supercomplexes in LV tissues from female hearts in both rats and humans (Fig. 9A, B). The importance of supercomplexes in the heart is underscored by the fact that in certain heart failure models, a reduction in supercomplexes was shown to be sufficient to reduce respiration capacity without detectable changes in individual complexes’ activities [46]. The assembly of supercomplexes is governed by several proteins, including COX7RP that brings together Complex I, III, and IV [3, 12]. The transcription of COX7RP is regulated in part by estrogen [33, 53], which prompted us to compare expression levels between sexes. Indeed, COX7RP was more abundant in rat and human female LV heart samples (Fig. 9C, D). Notably, COX7RP decreased in aged vs young female human LV heart tissues (Fig. 10A, B). Furthermore, ovariectomy significantly reduced COX7RP and supercomplexes in female LV tissue samples (Fig. 10C, D). Of note, it was previously reported that ovariectomy does not change diminished [Ca^2+^] uptake in female cardiac mitochondria [2]. In line with this report, our data shows no change in MCU levels in hearts from OVX rats vs shams and human tissue samples from old vs young females. Moreover, mito-Ca^2+^ measurements in VCMs from old rats showed that sex differences in mito-Ca^2+^ uptake and removal were still present despite loss of differences in intracellular Ca^2+^ handling and levels of mito-ROS (Fig. 7). Together, these data implicate loss of COX7RP as a key driver for increased mito-ROS production in low estrogen conditions.

Our in vitro gain- and loss-of-function experiments in H9c2 cells demonstrated a strong capacity of COX7RP to regulate respiration in these cells (Fig. 11). In VCMs from male rat hearts, COX7RP overexpression not only increased supercomplexes (Fig. 12), but also reduced mito-ROS and RyR2 oxidation, which led to increased SR Ca^2+^ load and reduced propensity for pro-arrhythmic SCWs (Fig. 14). Importantly, COX7RP KD in female VCMs led to entirely opposite results producing adverse effects on cytosolic Ca^2+^ cycling and ROS. Our results point to the possibility that downregulation of COX7RP [33, 53] and concomitant acceleration of mito-ROS production in the aging heart [15, 18] might contribute to abrupt increase in CVD risk in postmenopausal females. This would add COX7RP in females to the growing list of proteins involved in cardioprotection that show age-related changes in expression/activity, explaining loss of cardioprotection with age [8]. Interestingly, a recent study [32] ruled out any sex-related differences in infarct size and protection by ischemic pre-conditioning in minipigs, questioning the cardioprotection paradigm in premenopausal females. Our data may explain sexual dimorphism in some aspects of CVD risk, especially pathophysiology associated with arrhythmia and Ca^2+^ handling disturbances. However, these differences may not offer improved cardioprotection in response to severe irreversible ischemic injury resulting in necrosis and infarct.

## Conclusions

To summarize, our data highlight the fundamental differences in bidirectional SR-mitochondria communication in VCMs from heathy adult male and female hearts. Higher COX7RP-dependent ETC supercomplex assembly in female VCMs, combined with lower levels of mito-[Ca^2+^], underlies lower mito-ROS emission rates, reduced RyR2 oxidation, and lesser propensity for pro-arrhythmic spontaneous SR Ca^2+^ release under stress conditions. We conclude that sexual dimorphism in ETC organization and mitochondrial Ca^2+^ homeostasis may contribute to cardioprotection in healthy premenopausal females.

## Data Availability

All available data are incorporated into the article, raw data can be made avaliable upon reasonable request.

## Abbreviations

BN-PAGE: Blue native gel electrophoresis
COX7RP: Cytochrome c oxidase subunit 7A-related protein
CVD: Cardiovascular disease
ETC: Electron transport chain
ISO: Isoproterenol
MCU: Mitochondrial Ca^2+^ uniporter
NCX1: Na^+^ Ca^2+^ exchanger type 1
OVX: Ovariectomy
ROS: Reactive oxygen species
RyR2: Ryanodine receptor type 2
SCW: Spontaneous Ca^2+^ wave
SERCA2a: Sarcoplasmic endoplasmic reticulum Ca^2+^ ATPase type 2a
SR: Sarcoplasmic reticulum
VCM: Ventricular cardiomyocyte
